# The Role of Neutrophils and NETosis in Diseases: The Implications for Therapy

**DOI:** 10.1002/mco2.70770

**Published:** 2026-05-18

**Authors:** Zhen Ma, Qing Wang, Yusheng Zhang, Yang Li, Hongwei Wu, Hongjun Yang, Xianyu Li

**Affiliations:** ^1^ Beijing Key Laboratory of Traditional Chinese Medicine Basic Research on Prevention and Treatment for Major Diseases Experimental Research Center China Academy of Chinese Medical Sciences Beijing China; ^2^ State Key Laboratory For Quality Ensurance and Sustainable Use of Dao‐di Herbs China Academy of Chinese Medical Sciences Beijing China; ^3^ School of Life Sciences Beijing University of Chinese Medicine Beijing China; ^4^ School of Life Sciences Shanxi University Taiyuan China

**Keywords:** heterogeneity, immune clock, multiomics, NETosis, neutrophils

## Abstract

Neutrophils are the most abundant innate immune cells and are pivotal first responders in host defense, playing an important role in maintaining body homeostasis and regulating pathological conditions. Growing evidence indicates that neutrophils exhibit functional heterogeneity and participate in immune regulation through processes such as neutrophil extracellular trap formation (NETosis). These findings have expanded the traditional view of neutrophils as short‐lived effector cells. Nevertheless, how distinct neutrophil states are temporally coordinated during disease development, and how this coordination may be therapeutically exploited, remains insufficiently understood. In this review, we provide a comprehensive overview of neutrophil biology, focusing on functional heterogeneity, NETosis, and their roles in infectious diseases, autoimmune disorders, and cancers. We further discuss emerging therapeutic strategies targeting neutrophils, as well as advanced technologies that have enabled high‐resolution characterization of neutrophil states and functions. Building on the evidence above, the concept of “neutrophil immune clock” is proposed to describe the temporal changes in neutrophil‐mediated immune responses. A time‐resolved perspective on neutrophil responses may offer new insights into disease progression and support the development of neutrophil‐targeted strategies for disease prevention and therapy.

## Graphical abstract

Neutrophils secrete cytokines, express specific surface proteins, increase or decrease in cell count, and their ratio to lymphocyte count can be used as biomarkers to characterize collective status and disease progression. Neutrophils can monitor and track the body's health status, “Wei Bing” status, disease status, and different times of disease occurrence and development. So, neutrophils are the optimal carriers of the body's immune clock.

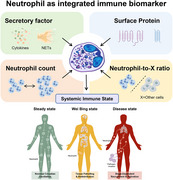

## 1 Introduction

Neutrophils are the most abundant and rapidly responding effector cells of the innate immune system and were long regarded as a short‐lived, homogeneous population with limited functional diversity. Over the past decade, this classical view has been fundamentally revised. Accumulating evidence now defines neutrophils as highly dynamic immune regulators characterized by pronounced heterogeneity, functional plasticity, and context‐dependent activities across tissues and disease states [1, 2, 3, 4]. Within the classical adhesion cascade, neutrophils undergo tightly regulated spatiotemporal trafficking between the circulation and inflammatory microenvironments, thereby shaping the initiation, amplification, and resolution of immune responses [3, 5, 6, 7]. In parallel, neutrophils deploy a broad repertoire of effector mechanisms, including phagocytosis, degranulation, cytokine amplification, and the release of neutrophil extracellular traps (NETs). NETs are web‐like structures composed of decondensed chromatin and granule‐derived proteins that contribute to both host defense and immune‐mediated tissue injury [8, 9]. NETs formation was initially linked to a distinct form of programmed cell death, leading to the concept of NETosis [10].

Beyond their well‐established effector functions, neutrophils exhibit striking temporal characteristics that add an additional layer of regulatory complexity. Both the abundance and phenotypic composition of circulating neutrophils fluctuate in a circadian manner, and population‐based studies have revealed robust diurnal variation in multiple hematological and immune parameters in humans [11, 12]. Experimental and clinical evidence further indicates that neutrophils respond dynamically to systemic rhythms and microenvironmental cues, integrating signals derived from circadian regulation, inflammatory mediators, and tissue context. Through processes such as migration, aging, NETosis, and immune‐regulatory amplification, neutrophils can act not only as drivers of inflammation and infection but also as sensitive cellular readouts of the host's physiological and pathological state [12, 13, 14]. Neutrophil behavior should be interpreted within a broader temporal framework that considers both physiological regulation and disease progression. Circadian and temporal features provide one important dimension of this integration. For example, the BMAL1–CXCL2–CXCR2 axis has been shown to govern circadian neutrophil aging, trafficking, and vascular protection, while consistent diurnal phenotypic shifts have been documented in both mice and healthy human volunteers, suggesting evolutionary conservation and potential translational relevance [14]. At the same time, neutrophil states also evolve over longer temporal scales during the transition from homeostasis to early immune imbalance, overt disease, and disease progression, indicating that neutrophil heterogeneity reflects not only circadian timing but also disease stage‐dependent dynamics.

Recent advances in single‐cell and spatial omics technologies have provided unprecedented resolution of neutrophil diversity across anatomical, physiological, and pathological contexts. Large‐scale single‐cell transcriptomic analyses have revealed that neutrophil states are organized along continuous trajectories rather than discrete categories, with preferential transitions observed under conditions such as health, inflammation, cancer, and infection [1, 4, 13]. Notably, integrating peripheral blood neutrophils into such reference atlases enables discrimination of aging, pregnancy, early‐stage tumors, infectious states, and active versus remission phases of disease based on their distribution patterns, highlighting the potential of neutrophils as dynamic indicators of host state [1]. In parallel, clinically accessible neutrophil‐derived parameters, including the neutrophil‐to‐lymphocyte ratio (NLR), have been validated as prognostic markers in multiple diseases, particularly solid tumors [15]. Incorporating temporal context, such as sampling time and circadian phase, may substantially enhance the interpretability and clinical utility of neutrophil‐based biomarkers [16, 17].

Given the central involvement of neutrophils and NETosis in diverse pathological processes, this review summarizes current advances in neutrophil heterogeneity and NETosis, with particular emphasis on spatiotemporal regulation, circadian influences, and disease‐associated dynamic transitions. We further discuss the molecular mechanisms and pathological functions of neutrophils and NETosis across inflammatory, autoimmune, infectious, and cancers. NIC as a framework linking time‐resolved neutrophil states to the transition from homeostasis to disease. NIC may guide disease monitoring, prognostic assessment, and time‐informed therapeutic strategies.

## 2 Multidimensional Profiling of Neutrophil Heterogeneity

**FIGURE 1:**
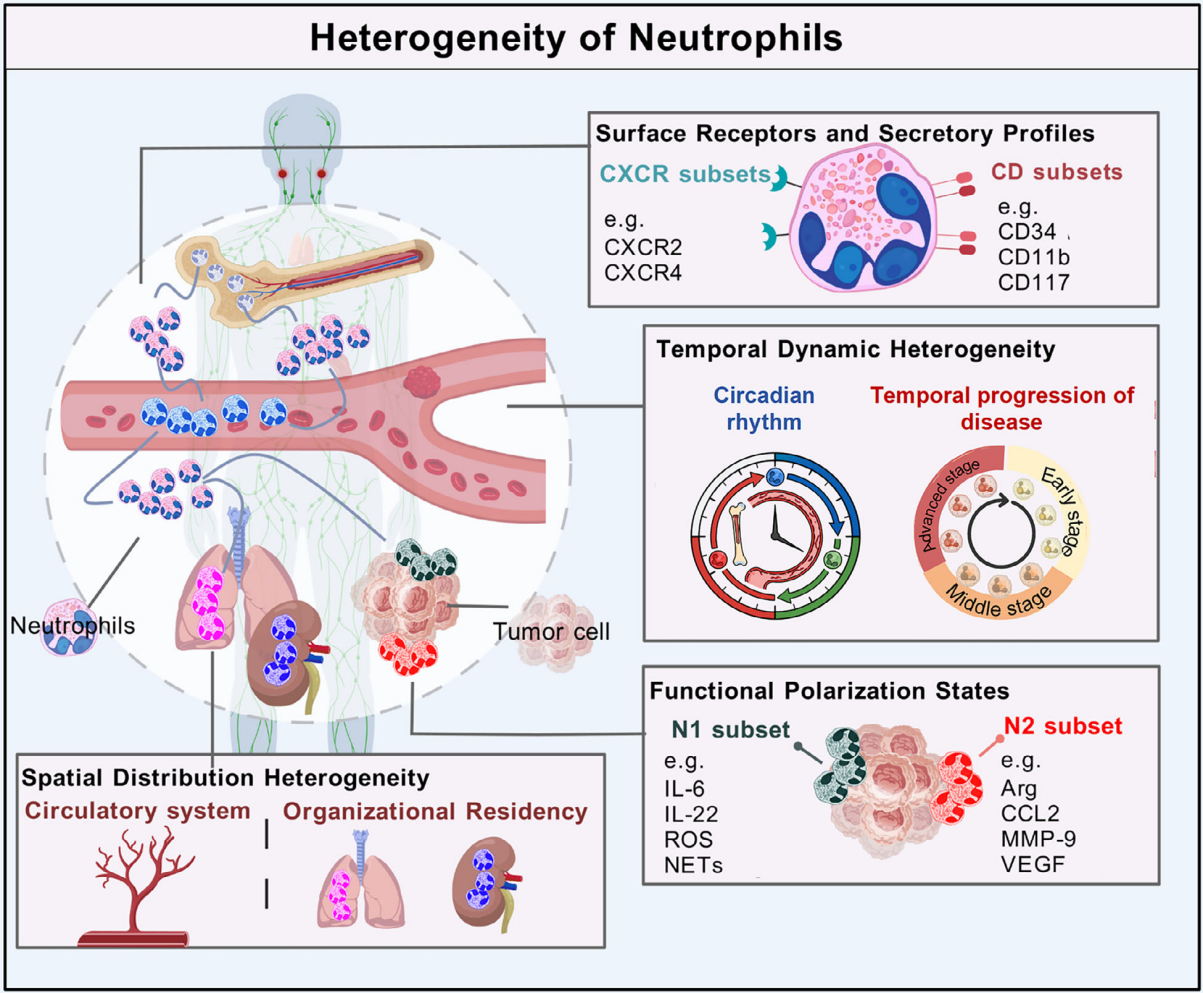
Heterogeneity of neutrophils. Neutrophil heterogeneity is manifested in multiple dimensions, including surface receptor expression and granule protein composition, functional polarization, temporal characteristics, and spatial distribution. The cell subtypes related to surface receptor expression and granule protein composition direction include CXCR family subtypes, CD family subtypes, and so on; functional polarization subtypes generally include N1 subtype and N2 subtype; heterogeneity of temporal characteristics refers to the heterogeneity of neutrophils in different states of the body's health and disease, or in different stages of the development of a unified disease; spatial distribution heterogeneity refers to the differences in neutrophils between the peripheral circulatory system and various tissues. *Abbreviations*: CXCR, C–X–C chemokine receptor; CD, cluster of differentiation; IL, interleukin; ROS, reactive oxygen species; NETs, neutrophil extracellular traps; Arg, arginase; CCL2, C‐C motif chemokine ligand 2; MMP‐9, matrix metalloproteinase‐9; VEGF, vascular endothelial growth factor.

Neutrophils exhibit profound heterogeneity. This heterogeneity manifests across multiple dimensions, including surface receptor expression, granule protein composition, metabolic characteristics, and spatiotemporal distribution, and influences their roles in disease. Neutrophils are activated by signals generated at inflammatory sites, leading to differential exposure of surface markers, de novo synthesis of cytokines, or polarization into distinct morphological/functional states. These defining features are commonly used to designate neutrophil subsets, as illustrated in Figure 1 [18, 19]. In this section, the current views on neutrophil heterogeneity are summarized, with special attention to the mechanisms of circadian rhythm, time, and spatial differences, which are the basis for supporting its functional plasticity.

### 2.1 Phenotypic Plasticity and Heterogeneity Classification

#### 2.1.1 Surface Receptors and Secretory Factors

Discussing surface receptors and secretory profiles requires defining key concepts. Cytokines refer to signaling proteins secreted extracellularly, including: chemokines (e.g., CXCL family), interferons (e.g., IFN‐α), interleukins (e.g., IL‐6), tumor necrosis factors (e.g., TNF‐α), and colony‐stimulating factors (e.g., GM‐CSF, G‐CSF). Neutrophil subsets can be classified based on the expression of surface receptors, such as chemokine receptors (e.g., CXCR family) or membrane proteins (e.g., CD molecules). As receptors or core components for chemokine CXCL ligands, CXCR subtypes exhibit distinct roles in neutrophil development. In neutrophil development, CXCR2 subsets typically represent mature cells, whereas CXCR4 subsets correspond to aged neutrophils [20]. Neutrophil retention in the bone marrow is regulated by coordinated CXCL12–CXCR4 signaling, with CXCR4 also mediating NETs formation [21, 22]. Conversely, CXCR2 and its ligands direct neutrophil egress from the bone marrow and critically govern pathological neutrophil migration and tissue infiltration [23, 24, 25, 26, 27]. Another major category of surface receptor subtypes belongs to the CD molecule family, which comprises transmembrane proteins including receptors, adhesion factors, and enzymes.

Neutrophils arise from granulocyte–monocyte progenitors (GMPs), which are commonly characterized by CD34^+^, CD38^+^, and CD45RA^+^ expression [28, 29]. GMP‐derived neutrophil progenitors then progress through a well‐defined developmental continuum (promyelocyte→myelocyte→metamyelocyte→band cell→segmented/mature neutrophil), accompanied by stepwise changes in surface phenotype: CD11b increases with late‐stage maturation, CD16b is acquired during terminal differentiation, and immature marrow neutrophils (metamyelocytes/bands) typically show intermediate CD11b and lower CD16b while retaining CD71 and CD117, which decline as cells become fully mature [5, 6, 28, 30]. Under steady‐state conditions, terminally differentiated neutrophils are released from the bone marrow into the bloodstream; once in circulation, they continue to undergo time‐dependent phenotypic remodeling, which contributes to neutrophil heterogeneity across sampling time points. In resting states, the CD177 glycoprotein (NB1 antigen), which is expressed exclusively on the neutrophil surface in CD177^+^ neutrophils, marks a subset associated with enhanced extracellular trap formation [20, 31, 32]. CD177^+^ neutrophils signal through β_2_‐integrin‐dependent pathways to coordinate activation‐mediated mechanisms and regulate human neutrophil migration [33, 34, 35]. This surface receptor‐based heterogeneity classification demonstrates high cross‐species conservation, establishing it as the preferred approach for comparative neutrophil research [36]. Importantly, neutrophils undergo progressive phenotypic remodeling in the bloodstream (aging), which is often reflected by changes in commonly profiled markers (e.g., reduced CD62L with increased CXCR4 and higher activation/adhesion signatures), and this process is tuned by environmental and systemic cues [37, 38, 39, 40].

Neutrophil subsets capable of secreting exosomes and extracellular vesicles play significant roles in immune regulation and disease progression. For instance, myeloperoxidase (MPO), a heme‐containing peroxidase predominantly expressed in neutrophils, catalyzes the formation of reactive oxygen intermediates in the presence of hydrogen peroxide and halides [41, 42]. MPO‐expressing neutrophil subsets play critical roles in microbial killing and represent important therapeutic targets in inflammation, while also serving as essential components of NETs [43, 44, 45]. Mature active MPO isolated from these neutrophils exists as a 145 kDa homodimer. Through binding to CD11b/CD18 integrins, it induces neutrophil activation and adhesion, thereby increasing leukocyte accumulation at inflammatory sites [46]. Neutrophil effector functions (degranulation, ROS production, cytokine release, and tissue‐injury versus host‐defense balance) have also been reported to show time‐of‐day dependence, consistent with circadian regulation of inflammatory magnitude and tissue susceptibility across the day [38, 47, 48, 49]. Mechanistically, this temporal pattern can emerge from (i) rhythmic tissue and endothelial “entry signals” that gate when neutrophils are recruited and activated, and (ii) cell‐intrinsic timing programs that remodel the neutrophil proteome and responsiveness while in circulation [40, 50, 51, 52]. Descriptive catalogs of secreted molecules are most informative when interpreted together with circadian, sampling time, and recruitment phase, because identical mediator profiles may correspond to distinct activation states [40, 53, 54].

Beyond conventional surface markers and secreted mediators, major histocompatibility complex (MHC) represents a functionally informative marker class for annotating neutrophil states. By displaying intracellular or extracellular antigenic signals on the cell surface for T‐cell recognition [55, 56], MHC expression provides a molecular readout of immune surveillance and tissue compatibility [57] and can therefore reflect disease‐associated neutrophil activation contexts. MHC‐I, expressed on most nucleated cells [58], has been implicated in neutrophil‐relevant inflammatory pathologies. Antibody engagement of endothelial MHC‐I induces complement activation and vascular injury in the lung, promoting platelet sequestration and edema [58, 59, 60]. In hepatitis B virus‐related acute liver failure, loss of MHC‐I on reactivated hepatocytes activates cytotoxic NK cells and induces pyroptosis, leading to neutrophil accumulation and HMGB1‐driven pathological NETs formation [61]. MHC‐II expression, although classically restricted to professional antigen‐presenting cells, can emerge in disease‐associated neutrophil populations. In glioblastoma, a dendritic‐neutrophil hybrid population expressing neutrophil markers (CD66b/CD16) together with MHCII/CD83 exhibits antigen‐processing and presentation capacity, activates T cells, enhances CD8^+^ T cell cytotoxicity, and secretes proinflammatory and stem cell‐regulatory factors, collectively contributing to tumor control [62, 63, 64, 65, 66].

#### 2.1.2 Functional Polarization States

Neutrophil polarization refers to their differentiation into distinct functional phenotypes under varying microenvironments or disease conditions. This process primarily classifies neutrophils into N1 and N2 polarization types. Compared with N2 neutrophils, N1 neutrophils exhibit higher levels of reactive oxygen species and enhanced oxidative burst, increased chemotactic responsiveness, and elevated expression of NADPH oxidase (NOX) subunits. These characteristics establish N1 neutrophils as proinflammatory effectors in innate immune responses [67, 68]. Crucially, N1 neutrophils demonstrate potent antitumor activity, primarily mediated through their release of proinflammatory and immunostimulatory cytokines such as IL‐12, TNF‐α, CCL3, CXCL9, and CXCL10. This cytokine profile promotes the recruitment and activation of CD8^+^ T cells [69, 70]. Conversely, N2 neutrophils are typically characterized as an anti‐inflammatory/protumorigenic subtype. Driven by signals such as TGF‐β and IL‐10, they exhibit immunosuppressive properties [71, 72]. Through modulation of molecules including ACE, AGTR2, and NOS1, N2 neutrophils promote angiogenesis, tissue repair, or tumor progression [71, 72, 73, 74]. Single‑cell transcriptomic and proteomic studies reveal that neutrophils in different functional states exhibit significant divergence in key signaling axes. For instance, core pathways such as NF‑κB, PI3K–AKT, and IFN–STAT show distinct profiles in terms of activation intensity and duration between inflammatory and immunosuppressive neutrophil phenotypes [18, 20, 75, 76]. Additionally, the low‐density neutrophils (LDNs) subtype is abundantly present under pathological conditions and exhibits context‐dependent functions across diseases. In patients with heart failure or autoimmune diseases, LDNs demonstrate elevated expression of H3Cit and NETs, along with enhanced proadhesive capacity [77, 78]. Within tumor microenvironments (TMEs), LDNs exhibit impaired effector functions and immunosuppressive properties, in contrast to mature high‐density neutrophils [75]. Tumor‑associated neutrophils (TANs) and LDN subsets can sustain persistent NETosis and immunosuppressive functions by enhancing fatty acid oxidation and glutamine metabolism to maintain energy homeostasis [77, 78, 79]. In contrast, neutrophils generated following acute infection or acute respiratory distress syndrome (ARDS) often exhibit impaired mitochondrial function and reduced metabolic plasticity, directly compromising their chemotactic and phagocytic capacities [80]. Epigenetic regulation further shapes the temporal dimension of neutrophil heterogeneity. Studies have revealed that systemic inflammation, hypoxia, and metabolic stress can induce lasting chromatin remodeling—such as alterations in H3K4me3 and H3K27ac modifications—at the stage of bone marrow neutrophil precursors, thereby preprogramming the inflammatory response threshold and lifespan characteristics of subsequently generated neutrophils [81].

### 2.2 Temporal Dynamic and Spatial Distribution Heterogeneity Classification

The classifications above primarily express neutrophil plasticity in steady‐state and pathological states. However, in actual expression, neutrophil heterogeneity is often more complex, potentially requiring synergistic markers of various surface receptors, molecular granules, and so on, to define different heterogeneous cell types.

Neutrophil function and phenotype are not static but exhibit significant dynamic heterogeneity across varying timescales. Neutrophil temporal heterogeneity is coordinated by circadian signals that regulate bone marrow egress, endothelial entry, and cell‐intrinsic programs shaping phenotype and function during circulation. These systemic rhythms also control leukocyte recruitment and stromal‐endothelial cues governing trafficking. [48, 49, 82, 83, 84, 85]. In parallel, rhythmic “promigratory” factor expression in endothelium and leukocytes creates lineage‐ and tissue‐specific trafficking rhythms, thereby altering which neutrophil states are most represented in blood versus tissues at a given time point [50, 86]. Finally, neutrophil‐intrinsic and environment‐tuned timing programs (including microbiota‐dependent tuning and programmed proteome remodeling) can shift activation thresholds and effector balance across the day, providing a mechanistic basis for time‐of‐day differences in inflammatory magnitude and tissue injury in disease models [37, 38, 39, 40, 51, 52, 53, 54]. These mechanisms indicate that neutrophil subsets defined by surface markers or effector profiles may be enriched at distinct circadian phases, underscoring the need to report sampling time and to stratify experiments by circadian phase to avoid time‐of‐day confounding. The temporal remodeling of phenotypes during development demonstrates strict time‐dependent characteristics in neutrophil production within the bone marrow, their release into circulation, and functional differentiation in peripheral tissues. During bone marrow maturation, neutrophil precursors undergo a differentiation cascade, this progression is marked by sequential expression of surface markers including CD11b and CD16, along with granule proteins such as MPO and elastase [76]. Following maturation, neutrophil release into circulation is governed by the CXCR4/CXCR2 signaling pathway: resting‐state neutrophils expressing high‐CXCR4 are retained in the bone marrow, while CXCR2 upregulation promotes their migration into circulation [87]. After entering the bloodstream, the surface of neutrophils gradually loses CD62L, while the expression of integrins such as CD11b/CD18 increases, marking their transition from a patrol state to an activated state, ultimately mediating the bone marrow homing and clearance of senescent cells through the CD62L^low^–CXCR4^high^ phenotype [13, 88]. It is noteworthy that neutrophils exhibit marked heterogeneity in lifespan. While conventional understanding posited a lifespan of merely 6–24 h, recent studies demonstrate that specific subsets, such as proangiogenic neutrophils, can survive within tissues for several days [32]. This difference in lifespan is closely linked to metabolic programming: short‐lived neutrophils rely predominantly on glycolysis, whereas the long‐lived subsets sustain energy production through fatty acid oxidation [89].

Circadian context is one of a unifying axis for neutrophil subset definitions. Many proposed neutrophil subsets are influenced by the timing of sampling and by the phase of neutrophil trafficking [40, 48, 49, 86]. Several surface receptors that are widely used to define neutrophil “subsets” are not static features but vary systematically with time‐of‐day and with a time‐in‐circulation program. In mammals, leukocyte trafficking and blood neutrophil counts oscillate across the 24‐h cycle, driven by coordinated circadian signals and local niche/endothelial programs [48, 59, 82, 83, 84, 86]. During circulation, circadian time‐dependent remodeling alters surface marker expression and functional readiness, such that cells sampled at different times of day may appear as distinct subsets if circadian stage is not considered. Mechanistically, circadian programs modulate the likelihood that neutrophils occupy particular phenotypic or functional states within defined temporal windows, through rhythmic endothelial adhesion molecule and chemokine landscapes as well as cell‐intrinsic timing programs [40, 50, 82, 83, 84]. Explicitly incorporating time‐of‐day as an annotation or experimental variable can therefore refine neutrophil subset definitions and help distinguish stable heterogeneity from transient, time‐dependent state transitions.

The functional phenotype of neutrophils also undergoes dynamic evolution throughout the course of disease. During acute infection or the early inflammatory phase, neutrophils exert potent bactericidal effects through the rapid release of ROS, antimicrobial peptides, and NETs. At this stage, they exhibit high expression of CD66b and CD177, along with a burst of proinflammatory cytokines such as IL‐1β and TNF‐α [90]. In the late stage of inflammation or TME, neutrophils can differentiate into immune suppressive subgroups such as N2‐type, manifested by upregulation of Arg1, PD‐L1, and IL‐10, which weaken adaptive immune response by depleting the necessary arginine for T cell activation or transmitting inhibitory signals [91]. Single‐cell transcriptome studies further reveal the temporal shift in neutrophils states in sepsis. Early neutrophils display an antimicrobial profile (e.g., Camp, Lcn2), whereas later subsets adopt a regulatory phenotype marked by immune checkpoint expression (e.g., Cd274, Vista) and tissue repair‐associated genes (e.g., Mmp9, Tgfbi) [20]. Additionally, the core circadian rhythm gene BMAL1 modulates neutrophil bone marrow egress rhythmicity via the CXCL2/CXCR2 axis, resulting in diurnal fluctuations in their antimicrobial function [52].

The spatial heterogeneity of neutrophils is dynamically regulated by their specific anatomical location and local microenvironment. In steady‐state tissues, neutrophils exhibit distinct functional partitioning across different organs. Under homeostatic conditions, neutrophils within the alveolar lumen display high expression of CXCR2 and CD11b for rapid pathogen clearance, whereas the interstitial subpopulation suppresses inflammation and promotes tissue repair via IL‐10 secretion [91, 92]. In the spleen, CD49d^+^ neutrophils in the red pulp enhance plasma cell differentiation through B‐cell activating factor, while the marginal zone subpopulation inhibits CD8^+^ T cell activity via PD‐L1. Additionally, Siglec‐F(+) neutrophils in the spleen release IL‐10 during sepsis, playing a critical role in immune regulation and suppression of excessive inflammation [93, 94]. This spatial heterogeneity is precisely controlled by local microenvironmental signals. Future research should integrate spatial transcriptomics and intravital imaging to dissect their dynamic differentiation mechanisms, as therapeutic targeting of specific subsets holds promise for novel disease intervention strategies.

The heterogeneity of neutrophils is not static but evolves over time, indicating that temporal regulation is an integral component of neutrophil biology. This temporal dimension provides a conceptual bridge to a novel paradigm of neutrophils as conveyors of immune timing information.

## 3 Molecular Mechanisms and Regulatory Networks of NETosis

NETosis refers to a form of neutrophil cell death characterized by the release of decondensed chromatin and granular proteins into the extracellular space, forming a web‑like structure that traps pathogens [95]. Studies have shown that the signaling mechanisms of NETosis are complex and diverse and can be activated via distinct pathways depending on the inducing factors [96, 97]. A deeper understanding of the core pathways of NETosis is crucial for elucidating its role in disease [98, 99].

### 3.1 Core Signaling Pathways of NETosis

The primary molecular mechanisms of NETosis include the classic ROS‐dependent pathway and non‐ROS‐dependent pathways.​

#### 3.1.1 ROS‐Dependent Pathway

The canonical NETosis pathway relies on NOX‐mediated ROS production and is often termed suicidal NETosis. Stimuli such as phorbol myristate acetate (PMA) or microbial agents can activate protein kinase C (PKC), elevate intracellular Ca^2^^+^ levels, and assemble the NOX complex to rapidly generate ROS [97, 100]. ROS acts as the central regulator of this pathway, activating neutrophil elastase (NE) and MPO, which translocate from granules to the nucleus to degrade histones and induce extensive chromatin relaxation [97, 101]. Meanwhile, ROS triggers calcium signaling and downstream effectors, promoting permeabilization of both the nuclear and plasma membranes [97]. Following nuclear envelope breakdown, nuclear contents spill into the cytoplasm, where NE and MPO further synergize to drive high‐level citrullination of histones, neutralizing positive charges on chromatin and facilitating decondensation [97, 102]. Peptidylarginine deiminase 4 (PAD4)‐mediated histone citrullination is a key step in chromatin loosening, and together with ROS‐induced histone carbamylation, it may enhance the pathogenic potential of NETs formation [99, 103]. At this stage, cyclin‐dependent kinases and receptor‐interacting protein kinase 3/MLKL are also activated, contributing to rupture of the nuclear and plasma membranes [97]. Ultimately, the pore‐forming protein Gasdermin D (GSDMD) is cleaved and activated by neutrophil proteases, punching pores in the plasma membrane to increase permeability, leading to cell lysis and the release of chromatin webs coated with antimicrobial molecules [100, 104]. GSDMD is a pore‑forming effector protein that promotes membrane perforation during NETosis. It is cleaved by activated NE to generate the active membrane‑perforating structure, which accelerates the release of NETs [100, 104]. Thus, suicidal NETosis captures and kills pathogens at the cost of cell death. It is noteworthy that various inflammatory mediators can induce NETs through this pathway: for example, IL‐8 significantly enhances NETs formation by activating downstream NOX and MAPK pathways via CXCR1/2 receptors [96, 105]; similarly, TNF‐α promotes ROS production and drives NETosis through membrane TNF–TNFR2 signaling, generating a pro‐NETosis neutrophil subpopulation in chronic inflammatory settings. Bacterial products, such as LPS activate NOX and proinflammatory MAPK via TLR4, can also induce mitochondrial ROS generation, and trigger mitochondrial DNA‐based NETs release [97, 99]. As such ROS‐dependent pathways involve cell lysis and intense oxidative stress, they are often associated with tissue damage and are considered sources of harmful factors in conditions like septic shock and autoimmune diseases [99].

#### 3.1.2 Non‐ROS‐Dependent Pathway

In addition to the classical ROS‐dependent pathway, neutrophils can also undergo vital NETosis. A defining feature of vital NETosis is that neutrophils remain viable after NETs release and retain functions such as chemotaxis and phagocytosis, supporting a role in localized immune defense [106]. This process relies primarily on PAD4‐mediated chromatin remodeling to enable NETs release while preserving plasma membrane integrity [97, 102]. PAD4 is a core enzyme in NETs formation across both ROS‐dependent and ROS‐independent pathways, catalyzing histone citrullination and promoting chromatin decondensation. Accordingly, loss or inhibition of PAD4 markedly reduces NETs production and alleviates disease phenotypes in mouse models of lupus and pulmonary fibrosis [97, 104]. NE further contributes to NETs formation by degrading histones and generating oxidative products, together driving chromatin decondensation and membrane destabilization; impairment of either process suppresses NETs release [97, 99]. Nevertheless, NETs generated through this pathway may still contain immunogenic self‐components, including oxidized mitochondrial DNA [99, 103]. These NET‐derived molecules can be sensed by innate immune receptors, most notably the cytosolic DNA‐sensing cGAS–STING pathway. Oxidized mitochondrial DNA within NETs has been shown to activate cGAS–STING signaling in macrophages and dendritic cells, inducing Type I IFN responses and contributing to autoimmune pathology, including systemic lupus erythematosus (SLE) [97, 99, 103]. While distinct stimuli may preferentially engage different upstream pathways, they ultimately converge on the activation of effector molecules such as NE and MPO, resulting in chromatin extrusion [97, 107].

**FIGURE 2:**
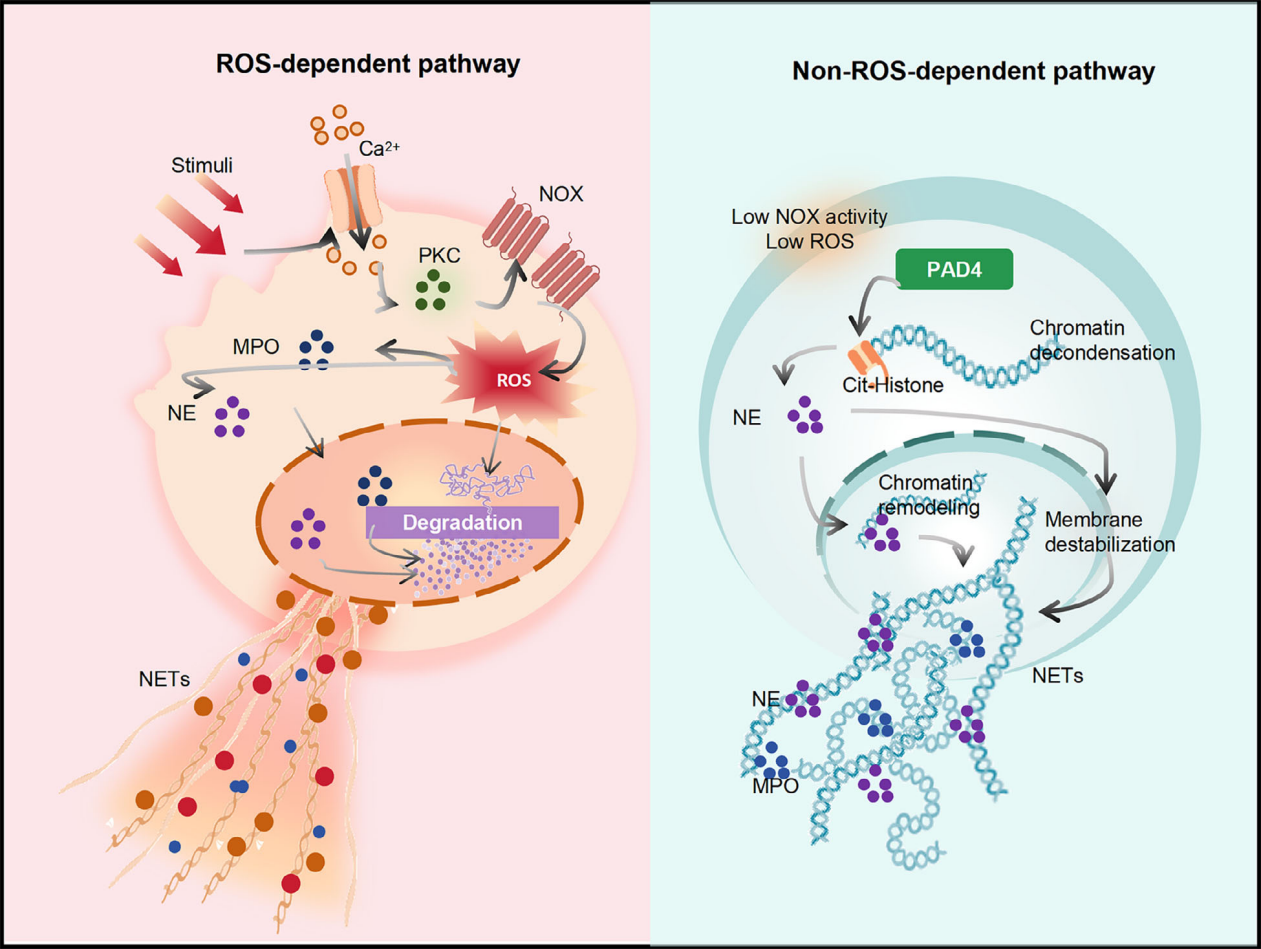
Distinct mechanisms of suicidal and vital NETosis. Neutrophils undergo suicidal NETosis through a ROS‐dependent pathway characterized by NADPH oxidase activation, extensive chromatin decondensation, membrane rupture, and cell lysis. In contrast, vital NETosis is driven primarily by PAD4‐mediated chromatin remodeling, allowing controlled NET release while preserving neutrophil viability. Despite distinct upstream signaling and membrane fate, both pathways converge on shared effector molecules, including PAD4, NE, and MPO, ultimately leading to chromatin extrusion. NETs generated via either pathway may exert antimicrobial functions but also contribute to inflammatory and immune dysregulation. *Abbreviations*: PKC, protein kinase C; NOX, NADPH oxidase; MPO, myeloperoxidase; NE, neutrophil elastase; PAD4, peptidylarginine deiminase 4.

NETosis is orchestrated by an interconnected signaling network in which ROS generation, chromatin decondensation, and membrane permeabilization represent key regulatory nodes. While NETs formation is essential for host defense, excessive or persistent NETosis can drive tissue damage and chronic inflammation. The dual nature of NETs underscores the importance of context‐ and timing‐dependent regulation. These features position NETosis as a critical mechanism in disease‐specific immune, which will be further explored in the context of human disorders and therapeutic interventions (Figure 2).

### 3.2 Metabolic Regulation of NETosis

During NETosis, cellular metabolic pathways regulate the timing of cytoskeletal remodeling, granule enzyme mobilization, and chromatin decondensation [108]. Metabolic status not only determines how NETosis occurs, but also shapes its impact in infections, cancer, and autoimmune diseases, including the extent of tissue damage. In this way, metabolism serves as a key link between molecular mechanisms and disease phenotypes. Among these pathways, glycolysis and lactate production provide an essential metabolic foundation for NETosis. Loss of the mitochondrial inner membrane fusion protein OPA1 reduces the activity of electron transport chain complex I, limits NAD^+^ supply, and in turn restricts ATP generation from glycolysis. This ultimately leads to a marked reduction in NETs formation [109]. In addition, inhibiting lactate dehydrogenase or altering the conformation of pyruvate kinase M2 can suppress NETs release. This regulatory mechanism is particularly relevant in autoimmune diseases such as antiphospholipid syndrome [110, 111].

The pentose phosphate pathway (PPP) plays a dual role in NETosis by supplying reducing power in the form of NADPH and generating nucleotide precursors. It is also closely linked to both ROS production and antioxidant defense. In neutrophils, the PPP provides reducing equivalents for NOX and supports the glutathione and thioredoxin systems, thereby helping maintain intracellular redox balance. During NETosis, sustained oxidative burst and chromatin remodeling increase the demand for both energy and reducing power, making the PPP a key metabolic branch that supports NOX activity and preserves redox homeostasis [108]. Mechanistically, the PPP promotes antimicrobial ROS production by increasing the supply of NADPH. At the same time, it works together with the glutathione system to limit irreversible cell damage caused by excessive oxidation. Through this dual regulatory role, the PPP helps control both the activation threshold and the duration of NETosis, and it may also influence whether cells enter a state of spontaneous or nonspecific NETs release [80, 108, 112].

Fatty acid signaling and glutamine metabolism together form a metabolic layer shaped by the microenvironment, translating changes in nutrient status into inflammatory responses. The accumulation of saturated fatty acids can activate the TLR4–MD2/ROS pathway, promote NETs formation, and enhance IL‐17‐driven inflammation. This process plays an important role in obesity‐associated psoriasis [113]. In contrast, glutamine shows anti‐inflammatory effects in sterile kidney injury. It reduces ROS production and NETs release, thereby limiting neutrophil recruitment to injured tissue. At the same time, glutamine can reprogram neutrophil gene expression, improve mitochondrial function, and support glutathione metabolism, which helps alleviate inflammatory damage [114].

The metabolic regulation of NETosis can be understood as three interconnected and parallel functional axes. First, glycolysis and lactate metabolism primarily determine energy supply and reaction dynamics. Second, the PPP supports redox balance by maintaining NOX‐dependent NADPH production. Third, fatty acid and glutamine metabolism sense and integrate nutrient signals from the pathological microenvironment, thereby shaping the activation threshold of NETosis and its inflammatory effects. These pathways form a multilayered regulatory network that governs the intensity and duration of NETosis, as well as its functional outcomes across different disease contexts.

### 3.3 Epigenetic and Transcriptional Regulation of NETosis

NETosis is a highly plastic process shaped by epigenetic states established in the bone marrow and transcriptional responses triggered by peripheral stimuli [115]. This regulation influences how NETosis occurs, as well as its duration and inflammatory impact in infections, cancer, and autoimmune diseases, linking intracellular control mechanisms to disease outcomes. At the bone marrow stage, specific epigenetic modifications can alter neutrophil effector functions, which in turn affects the magnitude and intensity of NETosis during later inflammatory responses. Studies in ARDS have shown that systemic hypoxia can induce histone H3 cleavage in neutrophil precursors and lead to a sustained loss of H3K4me3 at key inflammatory and antimicrobial gene loci. This results in impaired neutrophil function that can last for months [115]. More broadly, research on trained immunity shows that prior stimuli can reshape the distribution of H3K4me3 and H3K27ac, thereby altering how myeloid cells respond to subsequent challenges. At the same time, cellular metabolic status directly affects the availability of substrates required for epigenetic modifications. For example, the donor molecules for methylation and acetylation, as well as the cofactors needed for demethylation, are all generated through metabolic processes [116, 117].

During peripheral activation, neutrophil enhancer and promoter landscapes can be rapidly reshaped. Changes in H3K27ac, together with the binding of key transcription factors, drive stimulus‐specific transcriptional programs. After TLR8 activation, the genomic distribution of H3K27ac shifts quickly, accompanied by a redistribution of PU.1 and C/EBPβ binding sites. In regions that gain acetylation, OCT motifs are enriched and can be recognized by the newly expressed transcription factor OCT2 (POU2F2). OCT2 then works together with NF‐κB and AP‐1 to further enhance gene transcription, amplifying the expression of inflammation‐related genes. This process allows neutrophils, despite their short lifespan, to establish a relatively stable activated transcriptional state after stimulation [118]. Consistent with this, ChIP‐seq analysis of H3K4me3 shows that neutrophils display distinct transcription start site occupancy patterns under IL‐10 or TNF‐α pretreatment, or after LPS stimulation. These changes are accompanied by shifts in NF‐κB‐associated gene targeting. The transition of neutrophils between proinflammatory and anti‐inflammatory states depends not only on the surrounding cytokine environment, but also on the redistribution of epigenetic marks at promoter regions [119]. In disease settings, such rapid enhancer remodeling can amplify NETosis during infection, while in autoimmune or tumor environments it may lead to persistent and dysregulated activation.

Increased histone acetylation enhances the basal level of NETosis under resting conditions and further promotes NETs release upon NOX activation. Notably, this effect does not rely on additional ROS generation, but is primarily mediated through enhanced transcriptional initiation and elongation [120]. In contrast, zinc‐dependent histone deacetylases (HDACs) act as important facilitators of NETosis. Pharmacological inhibition of HDACs reduces NETs formation and alleviates inflammation in models of infectious pneumonia and septic shock [121]. Mechanistically, HDAC‐mediated deacetylation creates a chromatin environment that permits PAD4 activity in the nucleus, enabling the transition from a transcriptionally active state to a releasable chromatin configuration. Consistently, highly selective and reversible PAD4 inhibitors are sufficient to block NETs formation, elevating histone citrullination from a correlative marker to a functionally essential step in NETosis [122]. Noncoding RNAs and their associated regulatory networks further shape NETosis outputs under pathological conditions, forming a multilayered regulatory axis that spans bone marrow and peripheral compartments. In models of abdominal sepsis, deficiency of miR‐155 significantly reduces PAD4 expression and histone H3 citrullination, thereby limiting NETs formation in lung tissue and attenuating tissue injury [123]. Similarly, reduced levels of miR‐223 are associated with increased NETs formation in peripheral blood and exacerbated skin inflammation, suggesting that miRNA‐mediated regulation can translate local inflammatory cues into systemic NETosis responses [124]. In disease settings, these noncoding RNA‐dependent mechanisms not only contribute to inflammatory amplification but may also influence the organ‐specific distribution of NETosis and its impact on tissue damage. In addition, histone methylation and demethylation are broadly involved in transcriptional regulation during inflammation and tumor progression. Their role as long‐term modulators of myeloid cell functional states is increasingly recognized, and they may represent an important extension of NETosis‐related research [117, 125].

Epigenetic programming established in the bone marrow sets the baseline, while enhancer remodeling driven by peripheral stimuli determines the pattern of response. At the same time, the coordinated activity of HDAC and PAD4 provides the permissive conditions required for chromatin release, whereas noncoding RNAs fine‐tune this process. Together, these layers of regulation offer an important theoretical basis for developing therapeutic strategies that target NETosis.

### 3.4 Regulatory Checkpoints and Resolution of NETosis

The physiological role of NETosis depends on its timing, mode of execution, balance between amplification and restraint, and the efficiency of clearance. These steps together form a set of hierarchical regulatory checkpoints [126]. Initiation mainly depends on whether intracellular ROS and nuclear damage signals reach the threshold needed to trigger chromatin decondensation. ROS induces oxidative DNA damage and activates the DNA damage response, promoting chromatin relaxation and reorganization—an early and critical step in NETosis [127]. It also acts both as a signaling molecule and as a direct driver by altering DNA structure. Meanwhile, PAD4‐mediated histone modifications further facilitate chromatin remodeling, enabling the shift from a compact, transcriptionally active state to one suitable for release. Overall, ROS‐driven DNA damage provides the trigger, while PAD4 and related chromatin changes enable chromatin release [122, 127].

NETosis execution is characterized by precise spatial coordination between the granule system and membrane dynamics. Its core molecular mechanism relies on a cascade involving MPO, NE, and GSDMD. At the level of granules, the azurosome complex—enriched in MPO and NE and localized to azurophilic granule membranes—mediates the selective release of NE into the cytosol in a ROS‐dependent manner, independent of classical membrane fusion. Once released, NE degrades F‐actin and translocates to the nucleus, where it promotes chromatin decondensation. This establishes a functional axis linking granules to nuclear responses [128]. With respect to ROS spatial regulation, PMA‐induced NETosis requires not only extracellular ROS but also ROS generated within granules and processed by MPO. Inhibition of intragranular MPO activity or scavenging of these localized ROS is sufficient to completely block NETs formation, indicating that subcellular ROS localization itself serves as a critical checkpoint in NETosis execution [129]. At the level of membrane dynamics, GSDMD acts as an essential pore‐forming protein. Upon activation, it generates plasma membrane pores that provide the permeability required for the release of chromatin–antimicrobial protein complexes [130, 131]. From a pathophysiological perspective, excessive activation of this execution module is closely associated with tissue damage, thrombosis, and microvascular occlusion. Accordingly, targeted inhibition of MPO, NE, or GSDMD has emerged as a promising strategy to limit NET‐associated pathological injury.

Amplification of NETosis largely depends on feedback mechanisms, in which NETs themselves act as damage‐associated molecular patterns (DAMPs) and further enhance inflammation. After macrophages and other myeloid cells engulf NETs, NET‐derived DNA can enter the cytosol and activate the cGAS–STING pathway, leading to the production of Type I IFNs. At the same time, NE can further boost this signaling, forming a positive feedback loop [132]. This shows that NETs are not just end products, but can function as secondary signals that sustain and amplify inflammation. Under chronic inflammation or metabolic imbalance, this feedback becomes even stronger. For example, the accumulation of saturated fatty acids not only induces NETosis but also enhances IL‐17‐driven inflammation, which adds to NET‐mediated signal amplification [113, 132].

Termination of NETosis depends on efficient clearance mechanisms. Macrophages can actively engulf NETs, and this process is enhanced by DNase I pretreatment. The complement component C1q also acts as an opsonin to promote NET uptake. Importantly, NET clearance does not necessarily trigger proinflammatory cytokine release, which helps resolve inflammation and restore tissue homeostasis [133]. Beyond local clearance, neutrophil function is also regulated by circadian rhythms. The intrinsic clock protein BMAL1 drives CXCL2 expression and, through CXCR2, promotes aging‐like transcriptional and migratory features in neutrophils, while CXCR4 counteracts this effect. This rhythmic regulation helps balance antimicrobial defense with the prevention of excessive inflammation, making it an important systemic layer of NETosis control [134, 135]. In sepsis‐associated acute lung injury, BMAL1 in myeloid cells can further limit neutrophil recruitment and NETs formation by suppressing CXCL2 expression and the CXCL2/CXCR2 axis, suggesting that circadian factors can act as functional regulators of inflammation in disease settings [136].

In summary, the initiation of NETosis is jointly driven by ROS‐mediated DNA damage and repair together with PAD4‐dependent chromatin licensing. Its execution relies on a spatially organized machinery involving intragranular ROS, MPO/NE, and GSDMD. Amplification and restraint are regulated through the cGAS–STING pathway and cytokine networks, while termination depends on DNase‐mediated clearance and circadian control. Importantly, these checkpoints not only define the biological process of NETosis, but also map onto a set of potential therapeutic targets, providing a systematic framework for precision treatment of NETosis‐related diseases.

## 4 The Role of Neutrophils and NETosis in Diseases

Accumulating evidence implicates neutrophils and NETs in a broad spectrum of infectious inflammatory, autoimmune, and malignant diseases. Rather than acting as passive responders, neutrophils actively shape disease trajectories through their heterogeneous and temporally regulated functions. In this section, we discuss disease‐specific roles of neutrophils and NETosis.

### 4.1 Infectious Diseases

A better understanding of neutrophil‐mediated immune responses may help guide therapeutic interventions in infectious diseases, in which neutrophils exhibit bidirectional migration and exert both protective and pathological effects. Tuberculosis, a quintessential infectious disease, profoundly exemplifies the bidirectional and heterogeneous nature of neutrophils. On one hand, neutrophils directly control the pathogen through phagocytosis and antimicrobial molecules. On the other hand, they suppress T cell proliferation and IFN‐γ secretion, thereby weakening T cell responses [137, 138, 139]. Sepsis is a life‐threatening organ dysfunction caused by a dysregulated host response to infection. Neutrophils, mobilized from the bone marrow in increased numbers via chemokines such as G‐CSF and CXCL1/CXCL8, extensively infiltrate infection sites and exert critical defensive functions through pathogen phagocytosis and the release of antimicrobial peptides and ROS [140, 141, 142]. However, under the uncontrolled inflammatory state of sepsis, neutrophil function becomes profoundly dysregulated. This manifests as impaired chemotaxis leading to aberrant tissue migration, resulting in significant sequestration within microvessels and microthrombi formation. Overactivated neutrophils release elastase, MPO, and proinflammatory cytokines (e.g., IL‐1β, TNF‐α), directly damaging endothelial cells and parenchymal tissues [143, 144]. In sepsis, delayed neutrophil apoptosis, regulated by survival factors (e.g., GM‐CSF, LPS) and inflammasome signaling (e.g., NLRP3), prolongs their proinflammatory state. The subsequent failure to clear senescent neutrophils further promotes the release of DAMPs, sustaining a vicious inflammatory cycle [145, 146]. This constitutes a core mechanism driving the progression of sepsis toward multiple organ failure [147, 148]. Furthermore, proteases, ROS signals, and heterogeneous neutrophil‐derived mediators released by overactivated neutrophils no longer target pathogens exclusively. Instead, they launch indiscriminate attacks on host structures, including vascular endothelial cells, basement membranes, and lung parenchyma. This collateral damage is a key driver of tissue injury, edema, organ dysfunction, and ultimately, multiple organ failure in conditions such as ARDS, pneumonia, and chronic infections [149, 150].

### 4.2 Autoimmune Diseases

Monitoring neutrophil dynamics, including subset ratios, LDNs abundance, and NETs formation, may help assess disease severity and progression in autoimmune diseases. The core feature of autoimmune diseases lies in the loss of immune tolerance to self‐antigens, leading to pathological immune responses against the body's own tissues, cells, or components, ultimately causing tissue damage and dysfunction [151]. Neutrophils act as active regulators and key drivers in the initiation, amplification, and tissue injury of autoimmunity. They participate in autoimmune pathology through multiple mechanisms, with NETs formation being one of the most central [152, 153, 154].

**FIGURE 3:**
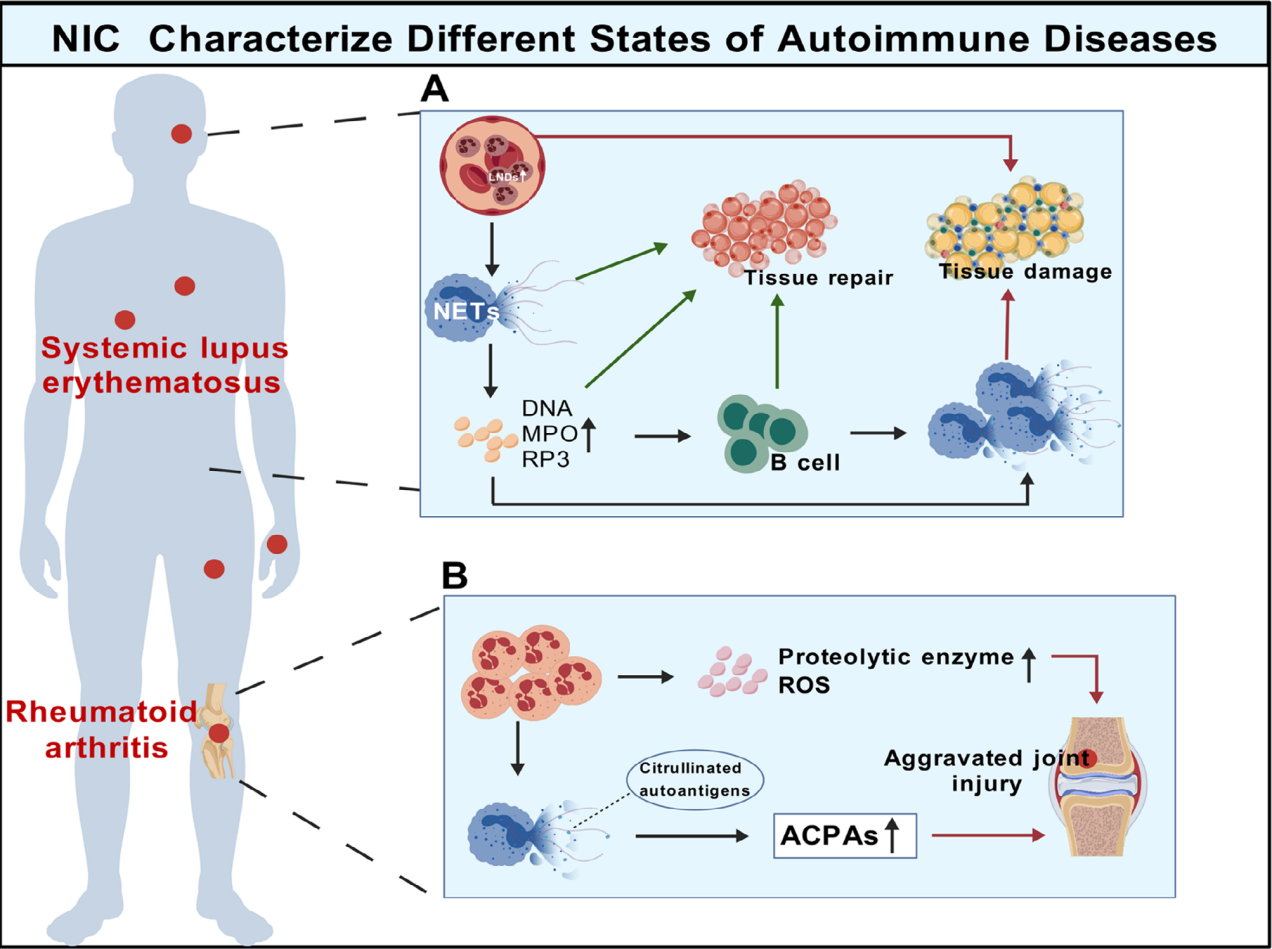
Dual role of neutrophils in autoimmune diseases. (A) In systemic lupus erythematosus, LDNs rapidly increase, producing a large number of NETs, which in turn release a large amount of NDA, MPO, RP3, and so on, stimulating B cells to promote tissue repair. At the same time, excessive NETs are also produced, causing damage to the tissue. (B) In rheumatoid arthritis, neutrophils perform tissue repair through NETs and other mechanisms, while ACPAs cooperate with excessive ROS and other molecules to cause further damage to the joints. *Abbreviations*: LDNs, low‐density neutrophils; PR3, proteinase 3; ACPAs, anticitrullinated protein antibodies.

In autoimmune contexts, NETs release abundant self‐antigens such as DNA, MPO, and PR3. These antigens can be recognized by autoreactive B cells, promoting autoantibody production [154, 155, 156]. In SLE patients, neutrophils not only exhibit quantitative changes but also display significant functional heterogeneity and abnormal activation states l (Figure 3A). This is characterized by an imbalance in the proportion of normal‐density neutrophils and LDNs in circulation [157, 158, 159, 160]. LDNs are markedly elevated in the peripheral blood of SLE patients. Their formation correlates closely with G‐CSF levels and disease activity. Compared with NDNs, LDNs typically possess stronger proinflammatory potential, produce higher levels of proinflammatory cytokines, express more self‐antigens, and exhibit resistance to apoptosis [159, 160, 161]. LDNs are considered the main source of pathological neutrophils in SLE, and a core pathogenic feature of LDNs is that they are highly prone to form NETs [78, 157]. Over released NETs have high immunogenicity, and their exposed self‐antigens can be recognized by plasma cell like dendritic cells, which is the core pathological feature of SLE [162, 163]. Additionally, SLE patients exhibit impaired NETs clearance due to reduced serum DNase I activity or the presence of DNase I inhibitors, resulting in persistent NETs and self‐antigens that exacerbate immune responses [164, 165]. Neutrophils play a critical effector role in the pathogenesis of rheumatoid arthritis (RA), driving synovial inflammation and joint destruction through multiple pathways (Figure 3B) [166]. Upon infiltrating the synovial joints, activated neutrophils release proteolytic enzymes and ROS from their granules, directly damaging cartilage and bone [167]. Simultaneously, neutrophils in the synovial fluid release NETs. These NETs contain citrullinated autoantigens, which serve as key targets for rheumatoid factor and anticitrullinated protein antibodies (ACPAs). The release of NETs directly exposes citrullinated autoantigens, thereby promoting ACPA production, amplifying inflammatory responses, and exacerbating chronic inflammation [167, 168, 169]. Furthermore, neutrophil quantification is clinically significant in RA. Studies demonstrate that the NLR and neutrophil percentage‐to‐albumin ratio correlate positively with RA severity [141, 142, 143]. Notably, NLR may serve as a cost‐effective, widely accessible independent predictor for RA diagnosis [170, 171, 172]. In autoimmune diseases, neutrophils have transitioned from being perceived as simple terminal effector cells to emerging as pivotal active participants in disease pathogenesis.

### 4.3 Cancers

Neutrophils, as core components of the innate immune system, exhibit a complex dual role in cancer development—functioning as either antitumor guardians (N1‐type) or protumor accomplices (N2‐type)—with their functional polarization finely regulated by cytokine networks in the TME [173, 174]. A time‐based analysis of neutrophil states may help clarify how neutrophils contribute to changes in the TME during cancer progression.

In terms of tumor promotion, TANs drive cancer progression through multiple mechanisms: they secrete factors such as VEGF, HGF, and IL‐8/CXCL8 to promote tumor cell proliferation and angiogenesis, paving the way for tumor invasion [175, 176, 177, 178, 179, 180]. Neutrophils form NETs that capture circulating tumor cells and activate metastasis‐related pathways, while suppressing T/NK cell function via Arg‐1 and ROS expression to establish an immunosuppressive microenvironment [181, 182, 183]. NETs further induce tumor metastasis by interacting with the transmembrane protein CCDC25 on cancer cells [175, 184, 185, 186]. Conversely, activated N1‐type TANs exert antitumor effects under specific conditions: directly killing tumor cells by cytotoxic substance release, enhancing targeted therapy through antibody‐dependent cell‐mediated cytotoxicity (ADCC), and recruiting/activating effector immune cells via chemokines like CXCL9/CXCL10 to coordinate antitumor immunity [181, 187, 188]. Neutrophils also play different roles in different cancers, reflecting their dual mechanisms. In pancreatic ductal adenocarcinoma (PDAC), increased neutrophil infiltration in the TME is associated with low survival rates in PDAC patients. TANs secrete large amounts of CCL5, thereby enhancing cancer cell migration and invasion. TAN subsets are negatively correlated with PDAC cytotoxic CD8^+^ T cell infiltration and promote T cell dysfunction [189, 190]. In addition, elevated levels of IL‐17, PADI4A, and TIMP1 can induce the generation of NETs and accelerate the development of PDAC [191, 192]. In breast cancer, NETs serve as key drivers of pulmonary metastasis. Tumor cells activate the neutrophil membrane protein PR3 through secreted proteases (e.g., CTSC), inducing IL‐1β release and NET formation [193]. Subsequently, NETs degrade TSP‐1, disrupting extracellular matrix integrity to directly facilitate cancer cell colonization [194]. Furthermore, in triple‐negative breast cancer, the oncogene c‐FOS promotes NETs generation by binding the PAD4, with the ROS–p38 signaling axis amplifying this process [195]. Tumor‐derived autophagosomes induce PD‐L1‐modified NETs via the HMGB1–TLR4–MyD88 pathway, suppressing T cell function and establishing an immunosuppressive microenvironment [196]. In addition, neutrophils undergo unique metabolic remodeling in the premetastatic microenvironment. In terms of lipid storage, lung interstitial cells induce neutrophils to store lipids by inhibiting adipose triglyceride lipase, which provides energy for metastatic cancer cells through macrophagy [197]. In terms of the mechanism of antiferroptosis, tumor infiltrating neutrophils highly express aconitine decarboxylase 1, which is activated by the GM‐CSF–JAK/STAT5 pathway to produce itaconic acid. It resists ferroptosis through the Nrf2 pathway and maintains the immunosuppressive function of TINs [198]. However, in terms of immune suppression polarization, Lin28B^+^ tumor cells release low let‐7s exosomes, inducing neutrophil transformation to N2‐type and high expression of PD‐L2, inhibiting antitumor immunity [199].

Neutrophils and NETs contribute to disease pathogenesis through multiple mechanisms. The timing, magnitude, and spatial distribution of neutrophil responses are key determinants of clinical outcomes, suggesting that temporal immunomodulation may be a potential target for future diagnostic and therapeutic strategies.

## 5 Neutrophil‐ and NETosis‐Targeted Therapeutic Interventions

Neutrophils and NETosis, as dynamically and temporally regulated immune components, are recognized as sensitive modulators whose functional states vary depending on the disease stage and timing, thereby sparking renewed interest in neutrophil‑targeted therapeutic strategies.

### 5.1 Neutrophils and NETosis as Therapeutic Delivery Vehicles

Neutrophils can be harnessed as therapeutic carriers through direct loading of drugs into live cells or by engineering biomimetic nanocarrier coated with neutrophil membranes. These strategies exploit the intrinsic chemotactic properties of neutrophils for targeted delivery, while conferring immune evasion and site‐specific targeting capabilities. [200, 201]. As the most abundant leukocytes in the blood, neutrophils represent an ideal therapeutic delivery platform. Their inherent ability to respond to inflammatory signals, adhere to endothelial cells, and transmigrate across endothelial barriers by undergoing morphological changes enables them to infiltrate inflammatory and tumor sites. This facilitates neutrophil‐mediated penetrative drug delivery and thereby enhances therapeutic efficacy [202, 203].

**FIGURE 4:**
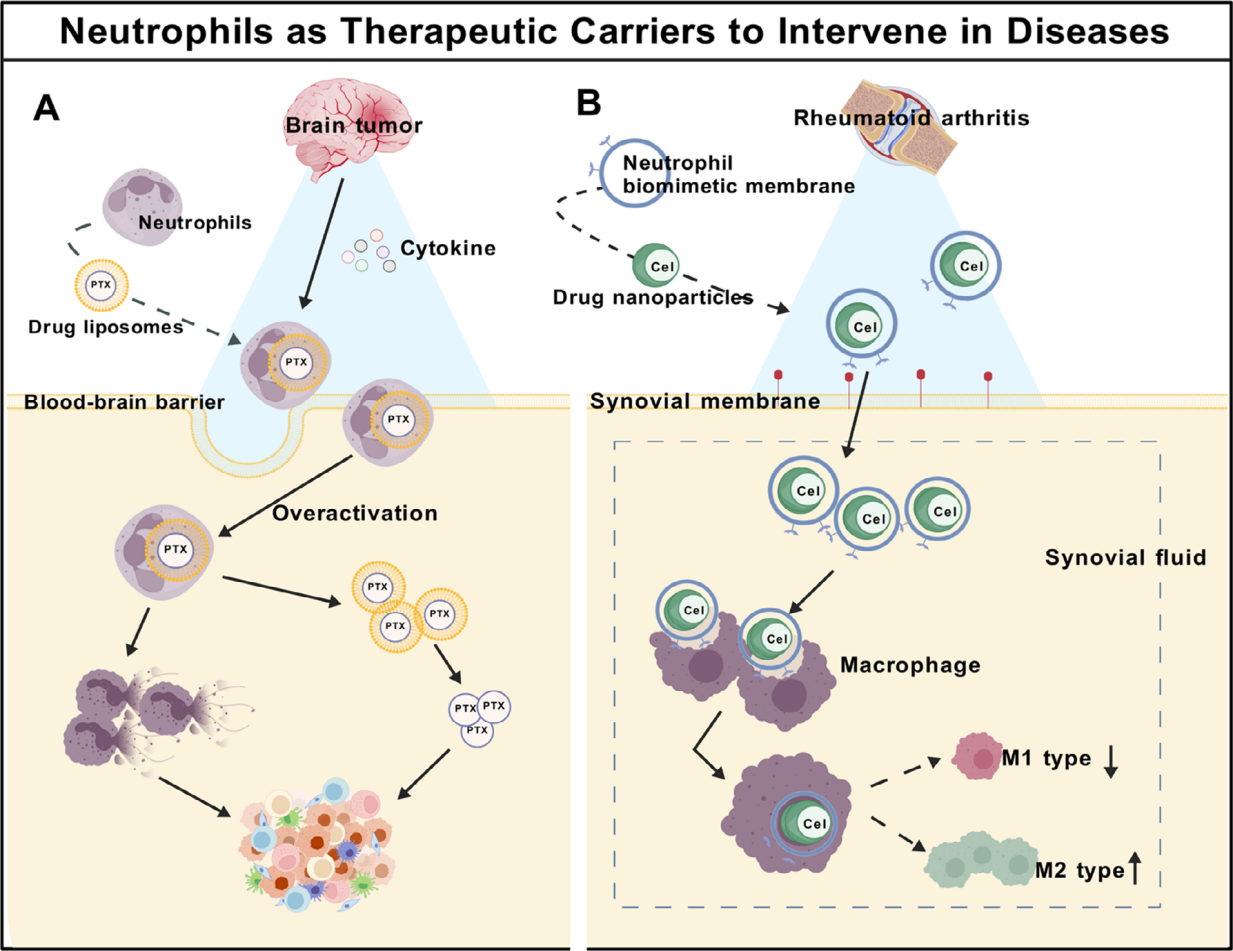
Neutrophils as therapeutic delivery vehicles. (A) In brain cancer, neutrophils encapsulate drug‐loaded liposomes, which over activate and release NETs and drug‐loaded liposomes after passing through the blood–brain barrier. Drug‐loaded liposomes target cancer cell sites for intervention therapy; (B) in rheumatoid arthritis, drug‐loaded nanoparticles wrapped in neutrophil membrane with specific antibodies can target macrophages and be engulfed by macrophages, stimulating macrophage differentiation and reducing M1 subtype and increasing M2 subtype. *Abbreviations*: PTX, paclitaxel; Cel, celastrol; M1, classically activated macrophage; M2, alternatively activated macrophage.

Multiple neutrophil‐vectored therapeutics have been developed. A representative example is PTX–CL/NEs, a novel autologous immune cell‐based delivery platform fabricated through simple incubation of neutrophils with paclitaxel (PTX)‐encapsulated glutamyl cationic liposomes (Figure 4A). PTX–CL/NEs induce neutrophils to cross the blood–brain barrier and enter the brain through inflammatory factors. PTX–CL/NEs are overactivated, releasing NETs and liposome PTX–CL. Subsequently, liposome PTX–CL effectively delivers PTX into tumor cells [204]. Alternatively, hollow TiO_2_‐coated long‐lasting luminescent nanoprobes ZGO@TiO_2_@ALP are internalized by neutrophils to form ZGO@TiO_2_@ALP‐NEs. Following intravenous injection, the inflammatory milieu of glioblastoma (GBM) recruits ZGO@TiO_2_@ALP‐NEs, which transmigrate through the blood–brain barrier via morphological changes, subsequently releasing their therapeutic payload to exert both therapeutic and recurrence‐preventive effects against GBM [205]. In the context of bone diseases, a drug delivery system utilizes neutrophils as “live transport vehicles” to precisely deliver therapeutics deep into the bone marrow. The key mechanism relies on drug‐loaded lipid nanoparticles being specifically conjugated with antibodies targeting neutrophil surface markers. This leverages neutrophils' inherent homing capacity to bone marrow and inflammatory sites, enabling active transport of nanoparticles across the bone marrow sinusoid barrier. Locally activated neutrophils then release the carried nanoparticles, achieving targeted drug release at disease foci [206]. A biomimetic neutrophil membrane‐coated F127 polymer nanocarrier modified with ApoA‐I‐mimetic peptides (R4F‐NM@F127) equipped with Celastrol (Cel) enables targeted drug delivery during RA therapy (Figure 4B). The neutrophil membrane coating confers synovitis‐targeting capability to the nanocarrier through high expression of adhesion molecules and chemokine receptors. Subsequently recruited into synovial fluid via IL‐8 chemotaxis, the system effectively suppresses synovial inflammation and alleviates joint damage by reprogramming macrophage polarization [207]. Intriguingly, neutrophils themselves can serve as potent weapons against cancer. NF‐κB through TNFR1 to induce neutrophil recruitment and activation within tumors. Anti‐CD40 therapy promotes neutrophil cytotoxicity and granulocytosis, while tumor‐targeting antibodies enhance tumor clearance via Fcγ receptor (FcγR)‐mediated ADCC. These therapeutic components activate complement through antigen‐antibody (Ag‐Ab) complex formation, inducing C5a generation. C5a signaling activates neutrophils via C5a receptor 1 (C5aR1) to produce leukotriene B4 (LTB4), thereby driving xanthine oxidase (XO) activity in the TME. XO‐generated ROS trigger oxidative damage and death of tumor cells, enabling neutrophils to eliminate diverse cancer cell types through this mechanism [181, 208].

### 5.2 Modulation of Neutrophil and NETosis Function

The immunosuppressive TME poses a major obstacle to effective immunotherapy. CD300ld plays a pivotal role in recruiting and mobilizing pathological neutrophils to tumor sites. These CD300ld‐guided neutrophils are specifically recruited to tumor loci where they orchestrate immunosuppression. Genetic knockout of CD300ld significantly reduces neutrophil infiltration into TMEs and effectively counteracts their immunosuppressive functions. Consequently, CD300ld small‐molecule inhibitors represent a potent neutrophil‐targeted strategy for tumor intervention [209]. The FDA has recently accepted the New Drug Application for brensocatib targeting noncystic fibrosis bronchiectasis (NCFBE). This inhibitor suppresses neutrophil serine protease (NSP) activation in bone marrow neutrophil precursors, thereby reducing protease burden in mature neutrophils and decreasing release of tissue‐destructive enzymes. Brensocatib fundamentally controls neutrophil‐mediated inflammatory storms, positioning it as the first mechanism‐targeted therapy for neutrophil‐driven diseases. This milestone signifies a paradigm shift from broad‐spectrum anti‐inflammatory strategies toward precise cellular targeting in inflammatory disease management [210].

Monoclonal antibody drugs (mAbs) constitute one of the earliest therapeutic categories developed based on neutrophils. Neutrophil‐targeting mAbs suppress tissue infiltration by blocking CD11b/CD18 integrins, with well‐established applications in neutrophil inhibition and antibacterial therapy [211, 212, 213]. CIT‐013, a novel high‐affinity monoclonal antibody targeting citrullinated histones H2A and H4, achieves dual effects by inhibiting NETs release and accelerating their clearance, currently advancing through Phase II clinical trials [214]. Furthermore, bispecific antibodies targeting Ly6G and CD11b have been developed to enable efficient, specific, persistent, and dose/time‐controllable neutrophil depletion in murine models. This depletion occurs primarily via macrophage‐mediated antibody‐dependent cellular phagocytosis (ADCP), demonstrating enhanced specificity and stability compared with conventional mAbs [215].

Beyond neutrophils themselves, NETs participate in autoimmune diseases and other noninfectious pathologies through neutrophil regulation, representing potential therapeutic targets for traditional Chinese medicines and natural products [216, 217]. NET‐associated DNA promotes tumor metastasis by binding to CCDC25 protein on cancer cells. A precise oligopeptide specifically blocks this CCDC25‐NETs‐DNA interaction, significantly inhibiting tumor cell metastatic capacity—a paradigm‐shifting approach utilizing peptides rather than conventional small‐molecule drugs [186]. Leveraging this specific interaction, researchers developed biomimetic CCDC25‐overexpressing hybrid membrane‐coated liposomes that target NETs‐associated pathologies. These liposomes substantially inhibit neutrophil recruitment and successfully suppress colorectal cancer liver metastasis [208]. Crucially, iron metabolism in neutrophils plays a vital role in NETs formation. A switchable peptide‐drug conjugate‐based iron nano‐chelator modulates neutrophil iron metabolism, inhibiting NETs generation and subsequent immunosuppressive functions, thereby offering an alternative therapeutic strategy targeting neutrophil iron regulation [218].

As summarized in Table 1, therapeutic strategies targeting neutrophils and NETosis are transitioning from mechanistic exploration toward a coherent clinical‐preclinical pipeline. Preclinical studies highlight the feasibility of exploiting neutrophils as active drug delivery vehicles, capitalizing on their intrinsic chemotaxis and transendothelial migration to achieve targeted delivery in inflammatory and TMEs, including across the blood–brain barrier. In parallel, modulation of neutrophil recruitment and effector functions, such as inhibition of pathological chemotactic signaling or suppression of neutrophil‐derived proteases, has emerged as an effective strategy to mitigate immunosuppression and tissue damage. Importantly, NETosis‐directed interventions are beginning to demonstrate clinical viability, exemplified by the histone‐targeting antibody CIT‐013 in Phase II trials and the neutrophil protease inhibitor brensocatib reaching regulatory review. These advances indicate that neutrophils and NETs are time sensitive and potentially druggable targets, and potentially for precision immunomodulatory therapies.

**TABLE 1:** Representative preclinical and clinical studies on neutrophil‐ and NETosis‐targeted therapeutic interventions.

Strategy category	Representative approach/agent	Core mechanism of action	Development stage	Indication/disease model	References
Neutrophil‐based drug delivery	Neutrophil‐mediated biomimetic delivery systems	Exploitation of neutrophil chemotaxis, transendothelial migration, and inflammatory homing to achieve targeted drug delivery	Preclinical	Cancer, inflammatory diseases	[200, 201, 202, 203]
	PTX–CL/NEs	Neutrophil‐mediated transport across the blood–brain barrier followed by NETs release and paclitaxel delivery within tumors	Preclinical	Glioblastoma	[204]
	ZGO@TiO_2_@ALP–NEs	Inflammation‐driven recruitment of neutrophil‐loaded nanoprobes enabling therapeutic delivery and recurrence prevention	Preclinical	Glioblastoma	[205]
	Antibody‐conjugated nanoparticle–neutrophil system	Antibody‐guided binding to neutrophils enables active transport and site‐specific drug release	Preclinical	Bone‐related diseases	[206]
	Neutrophil membrane‐coated biomimetic nanocarriers	Mimicking neutrophil adhesion and chemotactic properties to target inflamed synovium and remodel the immune microenvironment	Preclinical	Rheumatoid arthritis	[207]
Neutrophil activation therapy	Anti‐CD40 therapy combined with tumor‐targeting antibodies	Activation of neutrophils via the C5a–LTB4–XO–ROS axis, inducing oxidative tumor cell killing	Preclinical	Multiple tumor models	[181, 208]
Neutrophil modulation	CD300ld inhibitors	Blockade of pathological neutrophil recruitment, alleviating tumor‐associated immunosuppression	Preclinical	Cancer	[209]
	Brensocatib	Inhibition of neutrophil serine protease maturation at the bone marrow stage, reducing downstream inflammatory damage	Clinical (NDA accepted)	Noncystic fibrosis bronchiectasis	[210]
	Anti‐CD11b/CD18 monoclonal antibodies	Inhibition of neutrophil adhesion and tissue infiltration	Preclinical/early clinical	Inflammatory and infectious diseases	[211, 212, 213]
	Ly6G/CD11b bispecific antibodies	Controlled and specific neutrophil depletion via macrophage‐mediated ADCP	Preclinical	Inflammation and cancer models	[215]
NETosis‐targeted interventions	CIT‐013	Targeting citrullinated histones to inhibit NETs release and accelerate NETs clearance	Clinical Phase II	NETs‐associated diseases	[214]
	CCDC25–NETs DNA blocking peptide	Disruption of NETs–DNA interaction with tumor cell receptor CCDC25 to suppress metastasis	Preclinical	Tumor metastasis	[186]
	Natural products and traditional Chinese medicines	Modulation of neutrophil activity and NETs formation in noninfectious inflammatory conditions	Preclinical	Autoimmune and inflammatory diseases	[216, 217]
	Iron metabolism‐modulating nano‐chelators	Regulation of neutrophil iron homeostasis to suppress NETs formation and immunosuppressive functions	Preclinical	Tumor‐associated immunosuppression	[218]

*Abbreviations*: ADCP, antibody‐dependent cellular phagocytosis; ALP, alkaline phosphatase; C5a, complement component 5a; CCDC25, coiled‐coil domain‐containing protein 25; CD, cluster of differentiation; LTB4, leukotriene B4; NDA, new drug application; NEs, neutrophils; NETs, neutrophil extracellular traps; PTX, paclitaxel; ROS, reactive oxygen species; TiO2, titanium dioxide; XO, xanthine oxidase; ZGO, zinc gallate.

## 6 Advanced Technology Supports the Development of Neutrophils

Traditional static phenotypic classifications are increasingly insufficient to capture the heterogeneity, plasticity, and dynamic state transitions that characterize neutrophils across development, tissue contexts, and disease stages. Recent progress in genomics, transcriptomics, proteomics, and multiomics integration, provides a powerful methodological foundation for systematically interrogating neutrophil diversity over time and space.

### 6.1 Genomics

Genomics investigates the structure, function, evolution, and editing of entire genomes, which have been instrumental in identifying genetic determinants of neutrophil development and disease susceptibility [219]. Through genome‐wide association studies, genomics has revealed genetic variants influencing neutrophil counts, functions, and disease susceptibility [220]. Severe congenital neutropenia (SCN)—a monogenic disorder primarily caused by autosomal dominant mutations in the ELANE gene—disrupts neutrophil differentiation. CRISPR–Cas9‐mediated introduction of two single‐stranded DNA breaks on complementary DNA strands within the ELANE promoter TATA box suppresses ELANE mRNA expression. This editing effectively restores defective neutrophil differentiation in ELANE‐mutant CD34^+^ hematopoietic stem and progenitor cells both in vitro and in vivo, without compromising functionality of edited neutrophils [220, 221, 222, 223]. Similarly, mutations in genes such as HAX1 and GFI1 have been linked to SCN, underscoring the pivotal role of genomics in elucidating the genetic basis of neutrophil developmental defects [224, 225, 226, 227]. As a powerful tool, genomics has not only elucidated the genetic foundations and molecular regulatory networks governing neutrophil development and function but has also uncovered unprecedented heterogeneity and functional plasticity. This provides critical scientific insights for understanding neutrophils’ roles in health and disease, discovering novel diagnostic biomarkers, and identifying therapeutic targets.

### 6.2 Transcriptomics

Transcriptomics focuses on the complete repertoire of RNA molecules transcribed within specific cells, tissues, or organisms under defined temporal or physiological conditions, elucidating regulatory mechanisms of gene expression [228]. For instance, whole‐blood transcriptomic analysis of erythema nodosum leprosum (ENL) patients revealed significant enrichment of the CD177, a gene associated with neutrophil activation and degranulation, reinforcing the critical role of neutrophils in ENL pathogenesis [229]. Transcriptomics has also contributed significantly to deepening our understanding of neutrophils. Single‐cell transcriptomics systematically delineates the heterogeneous subpopulations and transcriptomic dynamics of neutrophils during maturation, differentiation, and fate determination under steady‐state and inflammatory conditions. Through isolation and scRNA‐seq analysis of murine bone marrow and peripheral blood neutrophils at steady state, five distinct bone marrow subpopulations were identified: three mitotically active subsets (G0, G1, G2) and two postmitotic mature subsets (G3, G4) [20]. In contrast, peripheral organs predominantly harbor three mature neutrophil subpopulations (PMNa, PMNb, PMNc) exhibiting distinct transcriptional signatures [214]. Single‐cell transcriptomics plays a significant role in cancer research. Integrated analysis of neutrophil scRNA‐seq data from 225 patient samples revealed ten distinct neutrophil clusters, each corresponding to unique immunophenotypes, chemokine signatures, and maturation states [58]. Transcriptomic profiling further demonstrated that HLA–DR^+^ CD74^+^ neutrophils correlate with improved prognosis across multiple cancer types, whereas VEGFA^+^ SPP1^+^ subsets associate with poorer outcomes [58]. Additionally, combined scRNA‐seq and spatial transcriptomics highlighted the pivotal role of TANs within the tumor‐infiltrating immune landscape of lip squamous cell carcinoma, while elucidating mechanisms underlying microwave thermo‐chemotherapy [230].

### 6.3 Proteomics

The sequencing of the human genome and numerous pathogen genomes has established a sequence‐based framework for proteomics development, thereby paving the way for its comprehensive application in neutrophil research [231]. Proteomic analyses reveal significant differential expression of four proteins in PMA‐induced NETs between RA and SLE patients: elevated RNASE2 in RA versus increased MPO, leukocyte elastase inhibitor, and thymidine phosphorylase in SLE. These protein alterations directly correlate with neutrophil activation status and cytokine storm formation, serving as both biomarkers for disease severity and therapeutic targets [232]. Sample source diversity further expands proteomic research dimensions. Blood proteomics directly reflects in vivo states by circumventing neutrophil activation artifacts from isolation procedures, making it ideal for dynamically monitoring neutrophil‐related protein biomarker changes during disease progression or therapeutic response [233]. For instance, comparative serum proteomics of centenarians versus younger controls identified >80 aging‐associated proteins, strongly indicating alterations in multiple pathways linked to healthspan and longevity [233]. Urine proteomics holds significant value in systemic diseases. Quantitative analysis of 1000 urinary proteins in 30 LN patients at the time of diagnostic renal biopsy and at 3, 6, and 12 months postbiopsy revealed multiple biological pathways, including chemotaxis, neutrophil activation, platelet degranulation, and extracellular matrix organization, and identified 237 urinary biomarkers associated with LN [234, 235]. Furthermore, feces also serve as a viable sample source for proteomic research. Proteomic analyses have demonstrated that fecal MPO correlates with the severity of alcohol‐associated hepatitis and can serve as a noninvasive prognostic indicator [236]. In summary, proteomics comprehensively delineates the protein landscape of neutrophils in health and disease, providing deep insights into their molecular mechanisms during processes such as infection defense, cancer progression, and autoimmunity. It identifies critical diagnostic markers and therapeutic targets, leveraging diverse sample sources including whole blood, urine, and tissue to advance translational medicine research.

### 6.4 Multiomics Integration

**FIGURE 5:**
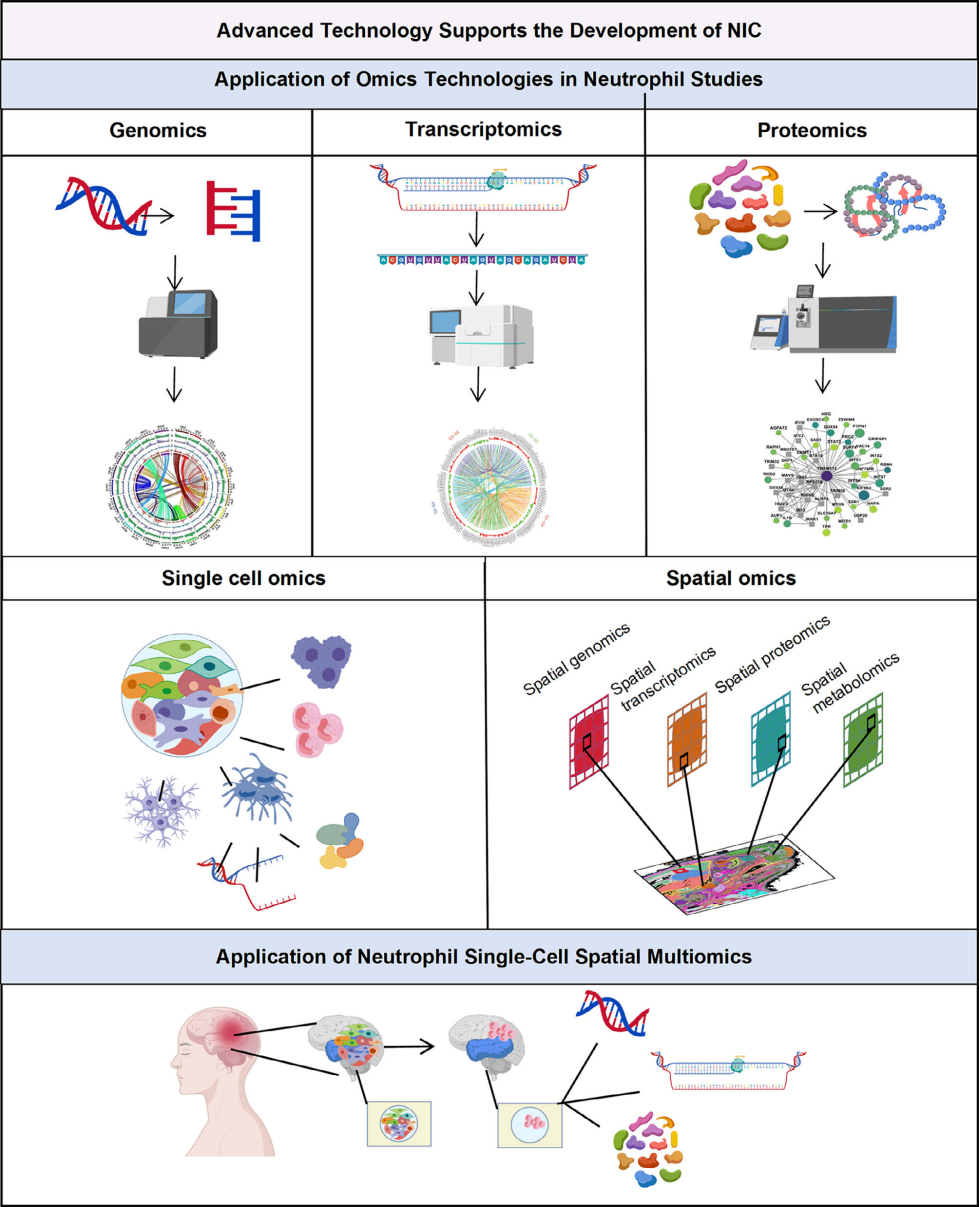
Advanced technology supports the development of neutrophil. The core of the advanced omics technology system applied to neutrophil research includes genomics, transcriptomics, and proteomics basic analysis. In addition, single‐cell omics techniques are used to analyze cellular heterogeneity, while cutting‐edge spatial omics techniques include spatial genomics, transcriptomics, proteomics, and metabolomics to locate the in situ distribution of specific molecules in the tissue microenvironment. The integrated application of single‐cell spatial multiomics provides strong technical support for in‐depth exploration of neutrophil function and regulation.

In recent years, the rapid development of multiomics integrative analysis, single‐cell resolution technologies, and spatial omics has profoundly revealed the heterogeneity, developmental trajectories, tissue‐specific functional states of neutrophils, and their dynamic interactions within the microenvironment. scRNA‐seq has been instrumental in overcoming technical barriers, systematically delineating the continuous differentiation lineages of neutrophils during bone marrow development across multiple species, including humans and mice. It has identified multistage states from precursor cells to mature neutrophils and uncovered previously unknown subpopulations within circulating neutrophils. These subpopulations exhibit distinct functional propensities under both steady‐state and inflammatory conditions [20, 237]. The application of spatial transcriptomics and spatially resolved proteomics has further expanded the research perspective from suspended cells to the tissue context. These techniques clearly map the precise spatial localization, abundance changes, and interaction networks of neutrophils with neighboring cells within complex tissues, such as the TME, sites of infection, and areas of injury repair [238, 239, 240]. This spatial information is crucial for understanding neutrophil function in specific pathophysiological contexts. For instance, neutrophils can adopt protumor or antitumor phenotypes within tumors, and their spatial distribution patterns significantly correlate with patient prognosis [75, 241]. Multiomics integrative analysis serves as the core strategy for in‐depth decoding of neutrophil complexity. By integrating data from scRNA‐seq, proteomics, metabolomics, and epigenomics, this approach enables the construction of more comprehensive molecular regulatory networks. It elucidates how these networks collectively shape neutrophil functional plasticity, lifespan regulation, and response to therapeutic interventions in contexts such as inflammation, infection, and cancer [242, 243]. For example, integrating metabolomic and transcriptomic data revealed an association between the unique arginine metabolism signature of TANs and their immune suppression function. TANs highly express Arg1 within the TME, leading to local arginine depletion and directly inhibiting T cell function [244, 245]. Furthermore, researchers combined single‐cell RNA sequencing and single‐cell proteomics of whole blood and peripheral blood mononuclear cells from COVID‐19 patients. This integration identified elevated HLA–DRhiCD11chi inflammatory monocytes bearing an IFN‐stimulated gene signature in mild COVID‐19. In contrast, severe COVID‐19 was characterized by the emergence of neutrophil precursors, serving as evidence of emergency myelopoiesis, alongside dysfunctional mature neutrophils and HLA–DRlo monocytes [246]. In summary, contemporary neutrophil research has entered an era characterized by multiomics approaches, single‐cell resolution, and high spatiotemporal resolution. This new paradigm is deciphering the neutrophil lifecycle, functional heterogeneity, and adaptation to tissue microenvironments with unprecedented depth and breadth. This progress opens promising prospects for the development of novel neutrophil‐based diagnostic markers and targeted therapeutic strategies (Figure 5).

The integration of genomics, transcriptomics, proteomics, and multiomics integration has markedly improved the resolution of neutrophil research. These approaches not only delineate the molecular and functional heterogeneity of neutrophils with unprecedented depth, but also enable reconstruction of developmental trajectories, tissue‐specific adaptations, and disease‐associated state transitions across time. From this perspective, changes in neutrophils states may reflect immune timing across physiological and pathological conditions. As these platforms continue to mature, their integration with clinical sampling and functional validation is expected to accelerate the translation of neutrophil‐based biomarkers and time‐informed therapeutic strategies.

## 7 Neutrophils Immune Clock

Based on the cumulative evidence summarized above, neutrophils exhibit pronounced heterogeneity in abundance, subset composition, and functional states across circadian, disease stages, and tissue contexts. These dynamic features suggest that neutrophil behavior is not static but evolves in a time‐dependent manner that reflects both physiological rhythms and pathological progression.

### 7.1 Conceptual Positioning and Supporting of the Neutrophil Immune Clock

Within this context, this paper proposes the concept of the NIC and clearly defines it as follows: NIC refers to the systematic, time‐dimensional mapping of a comprehensive dynamic atlas of the body's states—spanning health (including circadian rhythms), Wei Bing (a state in preventive medicine of traditional Chinese medicine, equivalent to sub‐health or predisease state), diseases (including different stages of diseases)—through the heterogeneity of neutrophils, comprising secretory factors, surface proteins, functional polarization, tissue specificity, and processes such as NETosis [247].

**FIGURE 6:**
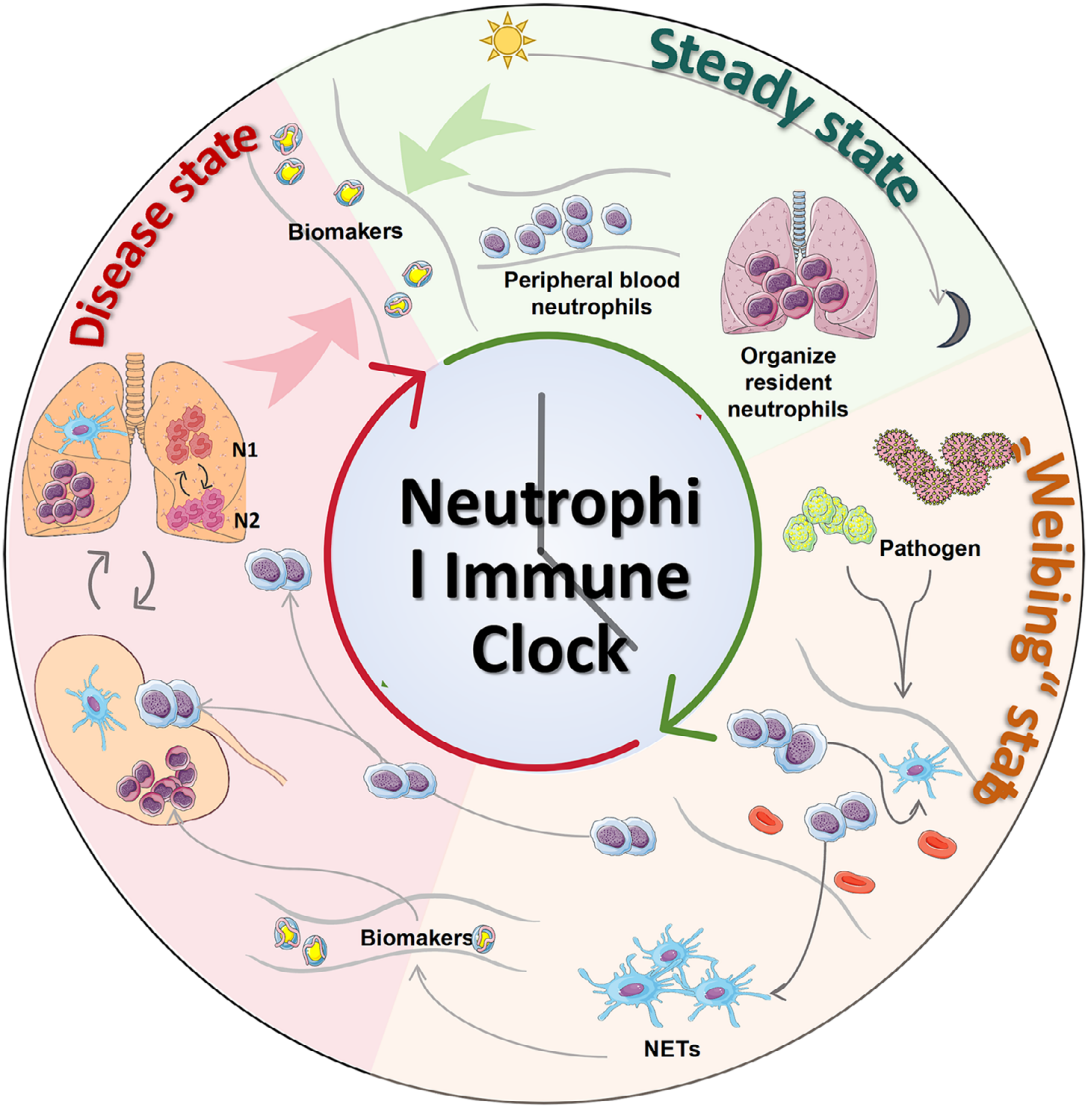
Neutrophil immune clock. By delving into the heterogeneity of neutrophils and utilizing various modern biological methods, based on neutrophils and their secreted substances, we aim to characterize the immune clock of neutrophils in the body, from healthy to “Wei Bing” to disease states, from early to mid to late disease development, as well as the distribution and synergistic effects of neutrophils among different tissues in different body states. *Abbreviations*: N1, antitumor/proinflammatory neutrophils; N2, protumor/immunosuppressive neutrophils.

The core objective of NIC is the systematic elucidation of neutrophil heterogeneity, functional plasticity, and molecular regulatory networks under physiological and pathological conditions, thereby providing novel biomarkers for disease diagnosis, stratification, and prediction of therapeutic response (Figure 6) [248]. NIC is a time‐dimensional, system‐level immunophenotyping framework that uses neutrophils and their heterogeneity to capture rhythmic or state‐dependent changes in neutrophil biology, which describes how neutrophils change over time across physiological and pathological contexts. Its “clock‐like” feature arises from coordinated shifts in neutrophil quantity, composition, and function, which together can form recognizable immune time fingerprints [52, 88]. These fingerprints may remain relatively stable within circadian constraints in healthy states, yet become disease‐ and stage‐specific under pathological perturbations. For example, the BMAL1–CXCL2–CXCR2 axis governs circadian neutrophil aging, trafficking, and vascular protection, while consistent diurnal phenotypic shifts observed in both mice and healthy human volunteers indicate conserved timing programs with translational relevance [14].

Recently, a large‐scale single‐cell transcriptomic research further support this time‐resolved view. By integrating neutrophil data across diverse anatomical, physiological, and pathological contexts, a global reference atlas revealed that neutrophil functional states are interconnected through continuous temporal trajectories, with preferential pathways emerging under conditions such as health, inflammation, and cancer [1]. Projecting peripheral blood neutrophils onto this atlas enabled discrimination of aging, pregnancy, early‐stage tumors, infections, and disease remission or activity, highlighting neutrophil distribution patterns as a dynamic readout of host state rather than fixed biomarkers [1]. These findings support the view that neutrophils states change in a time‐dependent manner and can be interpreted within the NIC framework.

### 7.2 Applications and Limitations of the NIC Framework

**FIGURE 7:**
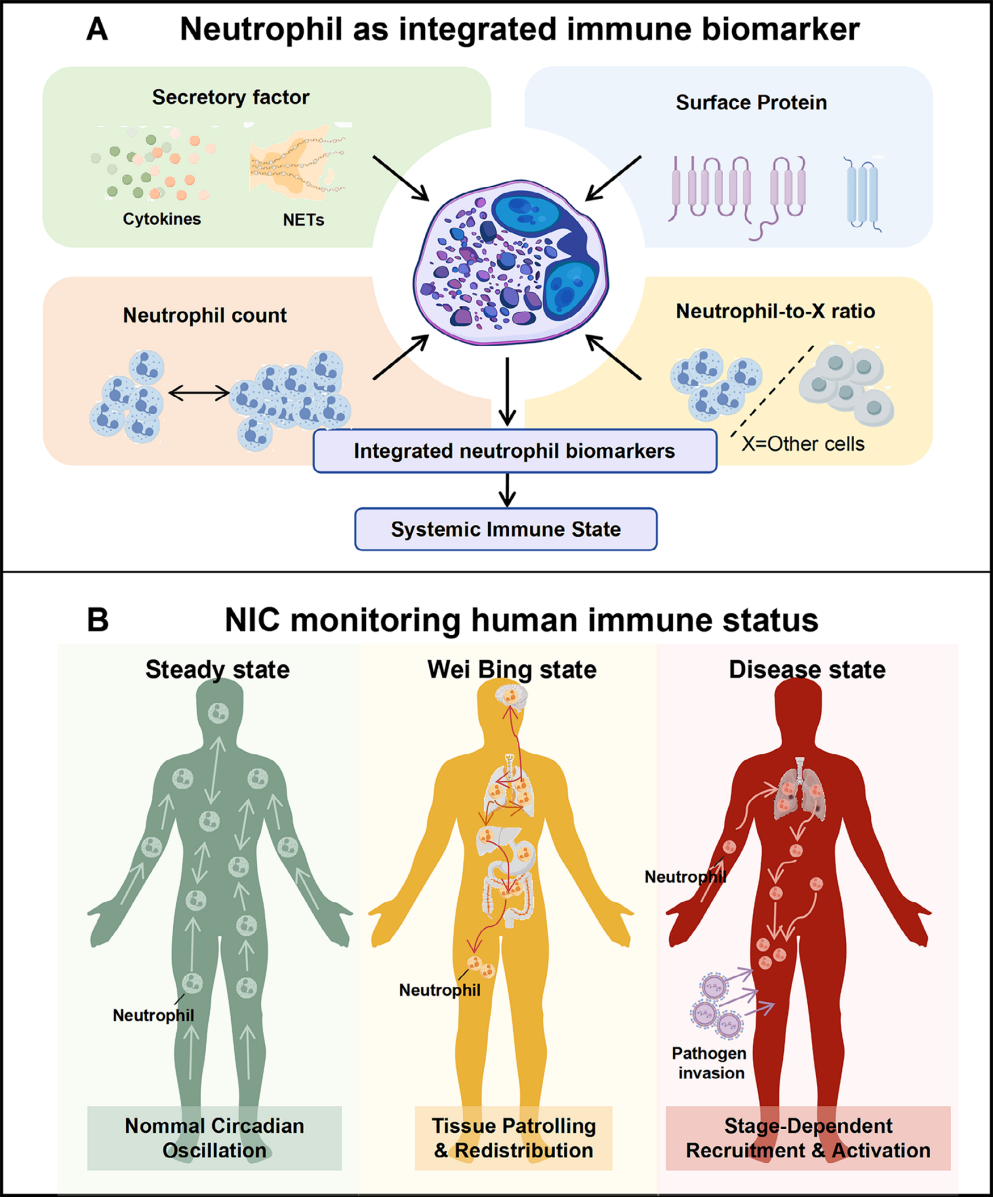
Neutrophils are the optimal carriers of the body's immune clock. (A) Neutrophils secrete cytokines, express specific surface proteins, increase or decrease in cell count, and their ratio to lymphocyte count can be used as biomarkers to characterize collective status and disease progression. (B) Neutrophils can monitor and track the body's health status, “Wei Bing” status, disease status, and different times of disease occurrence and development.

NIC is most informative when applied across the continuum from health to predisease susceptibility and overt disease. NIC framework highlights the potential of neutrophil‐based parameters as time‐sensitive biomarkers for disease monitoring (Figure 7).

#### 7.2.1 NIC Perform Monitoring and Defense Functions Throughout Physiological and Sub‐Health States

In a healthy state, neutrophils maintain a delicate balance: they are continuously released from the bone marrow, briefly remain in the bloodstream, and then migrate to tissues or undergo spontaneous apoptosis and clearance. This high turnover rate enables rapid responsiveness to internal and external environmental changes [5, 23, 249]. During aging, mature and immature atypical neutrophils can accumulate in blood, leading to decreased pathogen‐killing capacity and affecting susceptibility to infection and inflammatory conditions [250, 251]. When conventional clinical indicators still be normal, yet risk factors and pathological changes exist below diagnostic thresholds, it is a translational window that the early stage of homeostatic imbalance. This corresponds to the Wei Bing state, which individuals may be susceptible to overt disease development. Based on the characteristics of high abundance, short lifespan, high plasticity, and rapid responsiveness, neutrophils may exhibit subtle but measurable shifts earlier than abnormalities in traditional clinical indications, providing early, detectable signals of immune dysregulation. For example, the absolute neutrophil count (ANC) and its dynamic range can reflect hematopoietic reserve and bone‐marrow stress capacity. ANC persistently at the upper end of the normal range or mildly increased has been suggested to associate with increased risk of future cardiovascular events, and diabetes, consistent with occult low‐grade inflammation [252, 253, 254]. Other clinically accessible indicators, such as LDNs and NLR, have been widely associated with chronic inflammation, cardiovascular risk, autoimmune susceptibility, and cancer prognosis [170, 171, 172, 253, 254].

#### 7.2.2 NIC Perform Monitoring and Intervention Functions in Diseases

In established disease, neutrophils not only monitor disease status but also actively shape host responses. Their bidirectional roles are highly relevant for diagnosis and intervention strategies. In early infection, neutrophils rapidly recruit to infected sites, engulf pathogens, release MPO and other antimicrobial effectors, and form NETs through tightly regulated mechanisms, collectively constituting a first line of defense [255, 256, 257]. However, neutrophils can also transition from “guardians” to “destroyers” in specific contexts, a double‐edged nature that becomes evident during the pathological progression of infectious diseases [258, 259, 260, 261]. Neutrophils may leave the injury site and migrate through tissue interstitium toward vasculature; nevertheless, whether reverse migration is beneficial or detrimental remains to be fully elucidated [258]. Within this landscape, the NIC framework underscores the importance of interpreting neutrophil‐derived biomarkers in relation to sampling time, disease phase, and circadian context, rather than as isolated static measurements.

NIC‐guided clinical indicators also play a role in diseases. For example, systematic reviews and meta‐analyses have established that an elevated NLR is widely recognized as an important prognostic indicator in cancer [262, 263, 264]. Multiple clinical studies demonstrate that an elevated NLR in peripheral blood is an independent predictor of reduced overall survival in patients with various cancers, including pancreatic cancer, small cell lung cancer, and cervical cancer [262, 263, 264]. High NLR is also significantly associated with poor prognosis in nasopharyngeal carcinoma, gastric cancer, breast cancer, and endometrial cancer [265, 266]. Mechanistically, elevated NLR reflects enhanced systemic inflammatory responses and antitumor immunosuppression, such as suppressed T cell function, creating a favorable environment for tumor progression. High NLR is directly linked to tumor‐promoting neutrophil functions within the TME, including promoting angiogenesis, matrix remodeling, metastasis, and facilitating distant colonization by capturing circulating tumor cells via NETs [266]. In this way, NLR provides an immediately actionable entry point for NIC‐informed prognostic assessment, while motivating deeper mechanistic investigation into how neutrophil subsets evolve across disease stages.

### 7.3 Limitations and Practical Considerations of the NIC Framework

Although the NIC framework has potential for integration, it faces several limitations. Standardized definitions of neutrophil subsets remain incomplete, longitudinal human data are still limited, and precise in vivo manipulation of specific neutrophil states remains technically challenging. Moreover, excessive emphasis on temporal frameworks risks oversimplifying complex, context‐dependent immune processes. Addressing these challenges will require refined quantitative standards, mechanistic dissection of timing‐dependent transitions—particularly during early or transitional disease stages—and closer integration of single‐cell, spatial, and longitudinal clinical datasets [1, 4, 15, 16, 17, 79, 80, 81, 267, 268, 269, 270]. Viewed in this light, the NIC should be regarded as a complementary analytical lens rather than a definitive model, guiding future efforts toward time‐informed neutrophil‐ and NETosis‐targeted diagnostics and therapies.

## 8 Conclusions and Perspectives

Neutrophils constitute a highly abundant yet intricately organized immune cell population. Accumulating evidence demonstrates that neutrophil identity and function are dynamically regulated across multiple spatiotemporal dimensions. Epigenetic programming, phenotypic polarization, and temporal and spatial heterogeneity collectively shape a broad functional repertoire, enabling neutrophils to maintain immune homeostasis, respond to early immune imbalance or predisease states, and actively participate in disease initiation, progression, and resolution. Despite growing interest in neutrophil‐ and NETosis‐targeted strategies, their translation into disease prevention and clinical therapy remains constrained by several unresolved challenges. These include the limited ability to selectively manipulate specific neutrophil populations or functional states in vivo and insufficient understanding of the long‐term consequences of modulating neutrophil activity. Moreover, many investigations remain focused on isolated phenotypes, single effector functions, or specific disease models, limiting a systematic understanding of how neutrophil functional states and NETosis evolve over time and across tissue microenvironments. NETosis is often examined as an independent effector mechanism, while its integration with neutrophil state transitions, temporal regulation, and contextual cues remains incompletely defined. This fragmentation constrains efforts to translate time‐dependent neutrophil and NET‐based strategies into effective disease prevention and treatment. Addressing these challenges will be essential for advancing neutrophil‐based interventions.

For future research, the refined classification systems and quantitative standards to better capture neutrophil heterogeneity across physiological and pathological conditions would be first taken into consideration. Equally important is a deeper mechanistic elucidation of the molecular pathways that regulate neutrophil activation, functional plasticity, and NETosis, particularly during early or transitional stages of disease. In parallel, continued attention should be given to the advancement of targeted delivery strategies, as they are essential for selectively modulating pathogenic neutrophil subsets or NETosis without compromising protective immune functions. Moreover, the accelerated discovery and validation of neutrophil‐ and NET‐associated biomarkers are needed to facilitate early diagnosis, guide therapeutic stratification, and improve prognostic assessment. Overall, an integrated analytical approach is required to jointly characterize neutrophil subtypes and NETosis, define the temporal triggers and regulatory mechanisms underlying their functional divergence across disease stages, and establish dynamic monitoring strategies that support early warning, therapeutic evaluation, and optimization of intervention timing.

In this context, NIC can be used as a framework to link neutrophil heterogeneity, NETosis with disease progression. Rather than representing a single molecular pathway or autonomous immune pacemaker, the NIC emphasizes coordinated temporal changes in neutrophil abundance, subset composition, and functional states across the continuum from health to predisease and overt pathology. From a translational perspective, this framework highlights the potential value of time‐sensitive biomarkers and longitudinal assessment of neutrophil‐derived parameters for disease stratification, risk evaluation, and prognostic interpretation. Moreover, incorporating temporal principles into the design of neutrophil‐targeted interventions and chronotherapeutic strategies may help identify optimal therapeutic windows of inflammatory disorders, autoimmune diseases, cancers, and severe infections, thereby enhancing efficacy while limiting tissue injury [239, 240, 242].

The evidence reviewed indicates that neutrophils and NETosis are central mediators of immune regulation and disease pathogenesis and may represent targets for therapeutic intervention. Across diverse disease contexts, dysregulated neutrophil activation and excessive NETosis contribute may not only to tissue damage and disease exacerbation but also to treatment resistance and adverse outcomes. A deeper understanding of neutrophil heterogeneity, functional states, and temporal regulation will be critical for a more precise, time‐informed modulation of neutrophil and NETosis pathways. The integration of mechanistic studies with high‐resolution clinical data is expected to refine neutrophil‐based strategies for disease monitoring and intervention, ultimately improving therapeutic efficacy and safety.

## Author Contributions

*Data curation, conceptualization, methodology, and writing and original draft preparation*: Z.M. and Y.Z. *Review and editing*: X.L. and H.Y. *Visualization*: Q.W. *Supervision, project administration*, and *funding acquisition*: X.L. All authors have read and agreed to the published version of the manuscript.

## Conflicts of Interest

The authors declare no conflicts of interest.

## Ethics Statement

The authors have nothing to report.

## Data Availability

The original contributions presented in the study are included in the article.
